# Effects of a WHO-guided digital health intervention for depression in Syrian refugees in Lebanon: A randomized controlled trial

**DOI:** 10.1371/journal.pmed.1004025

**Published:** 2022-06-23

**Authors:** Pim Cuijpers, Eva Heim, Jinane Abi Ramia, Sebastian Burchert, Kenneth Carswell, Ilja Cornelisz, Christine Knaevelsrud, Philip Noun, Chris van Klaveren, Edith van’t Hof, Edwina Zoghbi, Mark van Ommeren, Rabih El Chammay

**Affiliations:** 1 Department of Clinical, Neuro and Developmental Psychology, Amsterdam Public Health Research Institute, Vrije Universiteit Amsterdam, the Netherlands; 2 Babeş-Bolyai University, International Institute for Psychotherapy, Cluj-Napoca, Romania; 3 Department of Psychology, University of Lausanne, Lausanne, Switzerland; 4 Institute of Psychology, University of Zurich, Zurich, Switzerland; 5 National Mental Health Programme, Ministry of Public Health of Lebanon, Beirut, Lebanon; 6 Department of Education and Psychology, Division of Clinical Psychological Intervention, Freie Universität Berlin, Berlin, Germany; 7 Department of Mental Health and Substance Use, World Health Organization, Geneva, Switzerland; 8 Department of Educational and Family Studies, Amsterdam Center for Learning Analytics, Vrije Universiteit Amsterdam, the Netherlands; 9 Country Office for Lebanon, World Health Organization, Beirut, Lebanon; 10 Psychiatry Department, Faculty of Medicine, Saint Joseph University, Beirut, Lebanon; International Organization for Migration, SRI LANKA

## Abstract

**Background:**

Most displaced people with mental disorders in low- and middle-income countries do not receive effective care, and their access to care has deteriorated during the Coronavirus Disease 2019 (COVID-19) pandemic. Digital mental health interventions are scalable when digital access is adequate, and they can be safely delivered during the COVID-19 pandemic. We examined whether a new WHO-guided digital mental health intervention, Step-by-Step, in which participants were supported by a nonspecialist helper, was effective in reducing depression among displaced people in Lebanon.

**Methods and findings:**

We conducted a single-blind, 2-arm pragmatic randomized clinical trial, comparing guided Step-by-Step with enhanced care as usual (ECAU) among displaced Syrians suffering from depression and impaired functioning in Lebanon. Primary outcomes were depression (Patient Health Questionnaire, PHQ-9) and impaired functioning (WHO Disability Assessment Schedule-12, WHODAS) at posttreatment. Secondary outcomes included subjective well-being, anxiety, post-traumatic stress, and self-described problems. A total of 569 displaced people from Syria with depression (PHQ-9 ≥ 10) and impaired functioning (WHODAS > 16) were randomized to Step-by-Step (*N* = 283; lost to follow-up: *N* = 167) or ECAU (*N* = 286; lost to follow-up: 133). Participants were considered to be lost to follow-up when they did not fill in the outcome measures at posttest or follow-up. Recruitment started on December 9, 2019 and was completed on July 9, 2020. The last follow-up assessments were collected in December 2020. The study team had access to the online platform, where they could see treatment arm assignment for each participant. All questionnaires were completed by participants online. Intention-to-treat (ITT) analyses showed intervention effects on depression (standardized mean differences [SMDs]: 0.48; 95% CI: 0.26; 0.70; *p* < 0.001), impaired functioning (SMD: 0.35; 95% CI: 0.14; 0.56; *p* < 0.001), post-traumatic stress (SMD: 0.36; 95% CI: 0.16; 0.56; *p* < 0.001), anxiety (SMD: 0.46; 95% CI: 0.24; 0.68; *p* < 0.001), subjective well-being (SMD: 0.47; 95% CI: 0.26; 0.68; *p* < 0.001), and self-identified personal problems (SMD: 0.49; 95% CI 0.28; 0.70; *p* < 0.001). Significant effects on all outcomes were maintained at 3 months follow-up. During the trial, one serious adverse event occurred, unrelated to the intervention. The main limitation of the current trial is the high dropout rate.

**Conclusions:**

In this study, we found that a guided, digital intervention was effective in reducing depression in displaced people in Lebanon. The guided WHO Step-by-Step intervention we examined should be made available to communities of displaced people that have digital access.

**Trial registration:**

ClinicalTrials.gov NCT03720769.

## Abstract 2

In a randomized controlled trial, Pim Cuijpers and colleagues evaluate the effects of the Step-by-Step guided digital mental health intervention on depression, impaired functioning, and other mental health outcomes among displaced Syrians living in Lebanon.

## Author summary

**Why was this study done?:**

- Depression is a major public health problem, and depression rates in people who are forcibly displaced are even higher.
- Effective treatment of depression is hardly available for displaced people.
- Digital mental health interventions may be a scalable solution for displaced people suffering from depression.

**What did the researchers do and find?:**

- We tested a digital tool for reducing depression in displaced people from Syria who live in Lebanon, based on an intervention for depression that was developed by WHO.
- We randomly assigned 569 displaced people to the digital tool or to a control group who only received usual care and found that people who received the digital tool were much less depressed after the intervention than the people who only received usual care. We also found important and statistically significant effects on anxiety, impaired functioning, post-traumatic stress, and well-being.
- Most effects persisted at 3 months after the intervention.

**What do these findings mean?:**

- We found that a guided, digital intervention was effective in reducing depression in displaced people in Lebanon.
- The intervention we examined can be an important tool to reduce depression in displaced people that have digital access.
- The findings should be interpreted with caution because of the large number of participants who dropped out of the study.

## Introduction

In 2022, an estimated 82 million people were forcibly displaced due to war, conflict, or persecution, including 21 million refugees [1]. The greatest contemporary crisis related to displaced people has been caused by the war in Syria, involving almost 6 million refugees [2]. Lebanon, with a total population of close to 7 million, currently hosts about 1.5 million displaced Syrians. Concurrently, Lebanon faces multiple other emergencies, including a collapsing economy, severe political turmoil, an explosion of neglected ammonium nitrate destroying large parts of the capital Beirut, and the impact of the Coronavirus Disease 2019 (COVID-19) pandemic [3].

During war, people often face multiple threats to life, loss of family, sexual and physical abuse, and lack of shelter or nutrition [4,5]. After fleeing for safety, displaced people experience continuing difficulties, including unmet basic needs, language barriers, uncertainty about the future, social isolation, and discrimination [4,6]. As a result, they are at risk of mental disorders, such as depression, anxiety, and post-traumatic stress [7]. It has been estimated that 22% of displaced, war-affected Syrians in Lebanon suffer from moderate to severe depressive symptoms [8].

Although depression and other common mental disorders are a leading cause of disability [9], the vast majority of displaced people do not receive treatment. This is especially true in low- and middle-income countries where only 1 in 27 people with depression are likely to receive evidence-based treatment [10] and where less than 1 per 1,000 displaced people seeks help from health services for common mental disorders [11].

Lebanon currently seeks to strengthen its mental health services. The National Mental Health Programme aims to scale up mental health care [12], but there have been limited resources, and there are recent challenges offering care safely during the pandemic and the political and economic crisis. One strategy to scale up services involves digital interventions, as research from high-income countries suggests that mobile apps can be effective [13]. Digital interventions can be either unguided or guided by a trained nonspecialist helper who supports participants in their use of self-help materials. While unguided interventions are less effective, guided self-help interventions are no less effective than face-to-face treatments [14]. Thus far, no studies on guided digital mental health interventions have been conducted in populations of displaced people in low- and middle-income countries.

A new WHO digital mental health intervention, “Step-by-Step,” based on behavioral activation to treat depression, can be delivered with guidance from nonspecialist helpers [15]. The current study examines the effects of guided “Step-by-Step,” compared to enhanced care as usual (ECAU) in displaced Syrians in Lebanon.

## Methods

### Design

This single-blind, 2-arm pragmatic randomized clinical trial examined the effects of a digital health intervention for depression compared with ECAU in displaced Syrians in Lebanon. The study was conducted together with an identical study in Lebanese citizens, which is reported elsewhere [16]. Ethical approval was received from the WHO Ethical Review Committee (ERC.0002797) and Saint-Joseph’s University, Beirut (CEHDF862). It was registered at ClinicalTrials.gov (NCT03720769). The trial protocol, an open pilot trial, and a feasibility randomised controlled trial (RCT) have been published [17–19].

This study is reported as per the Consolidated Standards of Reporting Trials (CONSORT) guideline (S1 Checklist).

### Procedures

We included participants meeting the following criteria: (1) Syrian displaced people; (2) above 18 years of age; (3) residing in Lebanon; (4) who understood and spoke Arabic or English; (5) had access to an internet-connected device; (6) had moderate or severe depressive symptoms (Patient Health Questionnaire [PHQ-9] ≥ 10) [20]l and (7) experienced functional impairment (WHO Disability Assessment Schedule-12 (WHODAS > 16)) [21]. Participants at imminent risk of suicide (based on a question on serious thoughts or a plan to end one’s life in the past month) were excluded. They received an on-screen message explaining that they may need additional mental health support. A national suicide hotline (phone number) was installed by a local NGO in Lebanon, which provides 24-hour support and referral to specialized care. People who respond “yes” to the PHQ question on suicide were informed about this 24-hour service, along with a list of mhGAP Interventions Guide trained primary health care facilities and were encouraged to seek help.

Recruitment of participants took place through advertising for the research project on several social media platforms, by posting and boosting posts on the official social media pages of the National Mental Health Programme on Facebook and Instagram. Additionally, outreach methods took place with the network of NGOs and UN agencies taking part in a mental health and psychosocial support taskforce whereby meetings were held in different regions with the Syrian community to introduce the project, followed by WhatsApp broadcasts that were sent by the organizations to their Syrian beneficiaries.

Interested participants could access the web version or download the mobile app, where information was given about the intervention and the study, including an animated video explaining key points. After completing informed consent and the baseline self-screening instruments, participants who met inclusion criteria were asked to complete additional baseline questionnaires. Informed consent was obtained through the online platform. Participants had to agree to 8 statements (e.g., I declare that I have read and understood the study information) by clicking the corresponding tick boxes. Otherwise, they were not able to proceed to the completion of the baseline questionnaires and to the intervention. As remuneration for completing all the questionnaires, users received $20 phone credit. Recruitment started on December 9, 2019 and was completed on July 9, 2020. The last follow-up assessments were collected in December 2020.

Participants were randomized to the intervention or ECAU, using a permuted block randomization with 1:1 allocation ratio within blocks of random length between 2 and 8. The randomization was handled by an algorithm for permuted block randomization that was built into the app and not accessible to the research team. The algorithm generated a random sequence of blocks with varying length. In each block, the number of seats for both groups was even, and the order was fully random.

The study team (i.e., coordinator and e-helpers) had access to the online platform, where they could see treatment arm assignment for each participant. However, as all questionnaires were completed by participants online, the fact that the study team was not blinded to treatment arm assignment did not have any effect on participants’ responses.

### Study arms

#### Intervention

Step-by-Step is a 5-session intervention, designed to treat depression through an internet-connected device [15]. It provides psychoeducation and training in behavioral activation through an illustrated narrative, with additional therapeutic techniques such as stress management, a gratitude exercise, positive self-talk, strengthening social support, and relapse prevention.

Step-by-Step includes 5 story sessions that are illustrated and audio recorded. Each session is divided into 3 smaller parts, which take altogether 20 minutes on average to read. Every session unlocks after 4 days of completing the previous session. This is to give enough time for the participant to practice the skills and exercises that they learned in the previous session. Users are recommended to complete 1 session (with all its 3 parts) every week. The program is thus meant to be completed over a period of 5 weeks to 8 weeks, accounting for delays. More information is in a paper about the development of the intervention [15].

The narrative was adapted to the local context, considering gender, linguistic, and cultural nuances among displaced Syrians [22]. It has a female and male version, each with 2 versions, 1 for married people with children and 1 for unmarried people. Participants can also choose the appearance of the character, broadly reflecting the main cultural groups in Lebanon (Lebanese, Syrians, and Palestinians). The intervention was provided as a hybrid app for iOS, Android, and web browsers using technical infrastructure developed by the Freie Universität Berlin [23]. Users who accessed the intervention received email or phone-based notifications, covering reminders of assessments due or upcoming, new sessions available, and gratitude for study participation. They could opt out of notifications any time.

Users of the intervention were supported by trained nonspecialists (“e-helpers”) who offered weekly phone or message-based contact with users to provide support (max. 15 minutes per week). E-helpers had a background in psychology or health, but had no previous experience in delivering mental health care. While the content of the intervention was delivered through the app, the e-helpers were trained to provide technical and emotional support to strengthen users’ motivation, to assess and refer participants at high risk of suicide, child abuse, or gender-based violence, and to support participants in acute distress, using preset protocols. E-helpers passed a competency test after the training to be involved in the trial. A treatment fidelity checklist was used, and 5% of the guidance calls and messages were rated. Training was delivered over 5 days with ongoing weekly group supervision—and, on demand, individual meetings—being provided by one local clinical psychologist.

Participants were considered inactive if they did not use the app for more than 3 weeks and did not respond to the e-helper reminders for 3 consecutive weeks. They then received an email informing them that the helpers would stop contacting them. Those who proactively conveyed that they would like to stop were also considered dropped out. The relevant e-helper then asked them if they want their data to be removed from the study and shared an exit survey with them by email to inquire about reasons for quitting.

#### ECAU

ECAU consisted of basic psychoeducation and referral to evidence-based care. Psychoeducation on depression and anxiety was delivered through the app or website. The text for the psychoeducational messages was taken from the first session of Step-by-Step to ensure identical information. After the psychoeducation, users received a list of primary health care facilities with nonspecialized staff trained in evidence-based mental health care [24].

### Outcomes

Primary outcomes were depressive symptoms measured by the PHQ-9 [20] and functional impairment measured by the WHODAS-12 2.0 [21] at posttreatment. The PHQ-9 is a 9-item instrument measuring severity of depression, with a cutoff score of ≥10 indicating moderate or severe depression, which has also been validated in Lebanon [25]. The WHODAS was used to measure functional impairment, as it provides a generic assessment of health and disability across 6 domains (cognition, mobility, self-care, getting along, life activities, and participation).

Secondary outcomes included subjective well-being, anxiety, and post-traumatic stress assessed by the 5-item WHO-5 Wellbeing Index [26]; the 7-item GAD-7 [27]; and the 8-item PTSD Checklist for DSM-5; PCL-5) [28], respectively. The Psychological Outcomes Profile (PSYCHLOPS) instrument was used to identify and rate self-described problems [29]. Satisfaction with the intervention was measured with the Client Satisfaction Questionnaire (CSQ-3), a measure of client satisfaction with mental health services [30]. A more extensive description of the measures can be found in the protocol paper [17].

Outcomes were measured at baseline, posttreatment (8 weeks after baseline), and follow-up (3 months posttreatment).

### Analyses

The RCT was designed to have >90% power with α = .05 to detect a 0.5 standardized mean difference (SMD) between the intervention and control group. Assuming 70% dropout [31,32], we planned to recruit 568 participants to show that the intervention was effective. We compared the intervention and control group on demographic and clinical characteristics with χ2 and variance analyses. The main outcomes were examined with intention-to-treat (ITT) analysis. Per-protocol analyses were secondary analyses.

For ITT analyses, regression models were estimated with treatment assignment status as principal predictor. To address potential bias concerns due to selective attrition, missing outcome observations were calculated using multiple imputation exploiting prescores and prespecified background characteristics (gender, age, education, and symptom severity). Given we consider continuous outcomes, multivariate normal regression imputation with an iterative Markov chain Monte Carlo method was used based on initial treatment assignment. The number of imputations required was determined using the 2-stage approach that ensures the replicability of SE estimates [33]. Performing this procedure indicated that at least 82 imputations were required, after which we decided on 150 imputations.

The prespecified covariates and baseline measurement of primary endpoint were added to the baseline model for improved precision. Effect sizes calculated are Hedges’ g by combining the multiple imputation estimation results using Rubin’s rules.

To examine the robustness of the results to differential nonrandom attrition across treatment arms, Random Forest Lee bounds (RFLBs) were estimated [34,35]. This bounding approach applies extreme worst- and best-case assumptions about the impact of differential selective attrition on the estimated effect for never-attriters. More details of these analyses are given in S1 Appendix.

To guide clinical interpretation, we calculated the proportion of participants who responded (>50% reduction of PHQ-9 symptoms) and completely remitted (<5 on the PHQ-9).

Concerns of multiple testing error were addressed by maintaining an experiment-wise type I error of 5%. To address potential heterogeneity, treatment effects were estimated for the 2 conditions (e.g., based on prescores). Finally, average treatment effects on the treated were estimated, and corresponding measures of clinically meaningful change and numbers needed to treat (NNT) were explored [36].

All analyses were conducted in STATA/SE 16.0 [37].

## Results

### Participants

**Fig 1:**
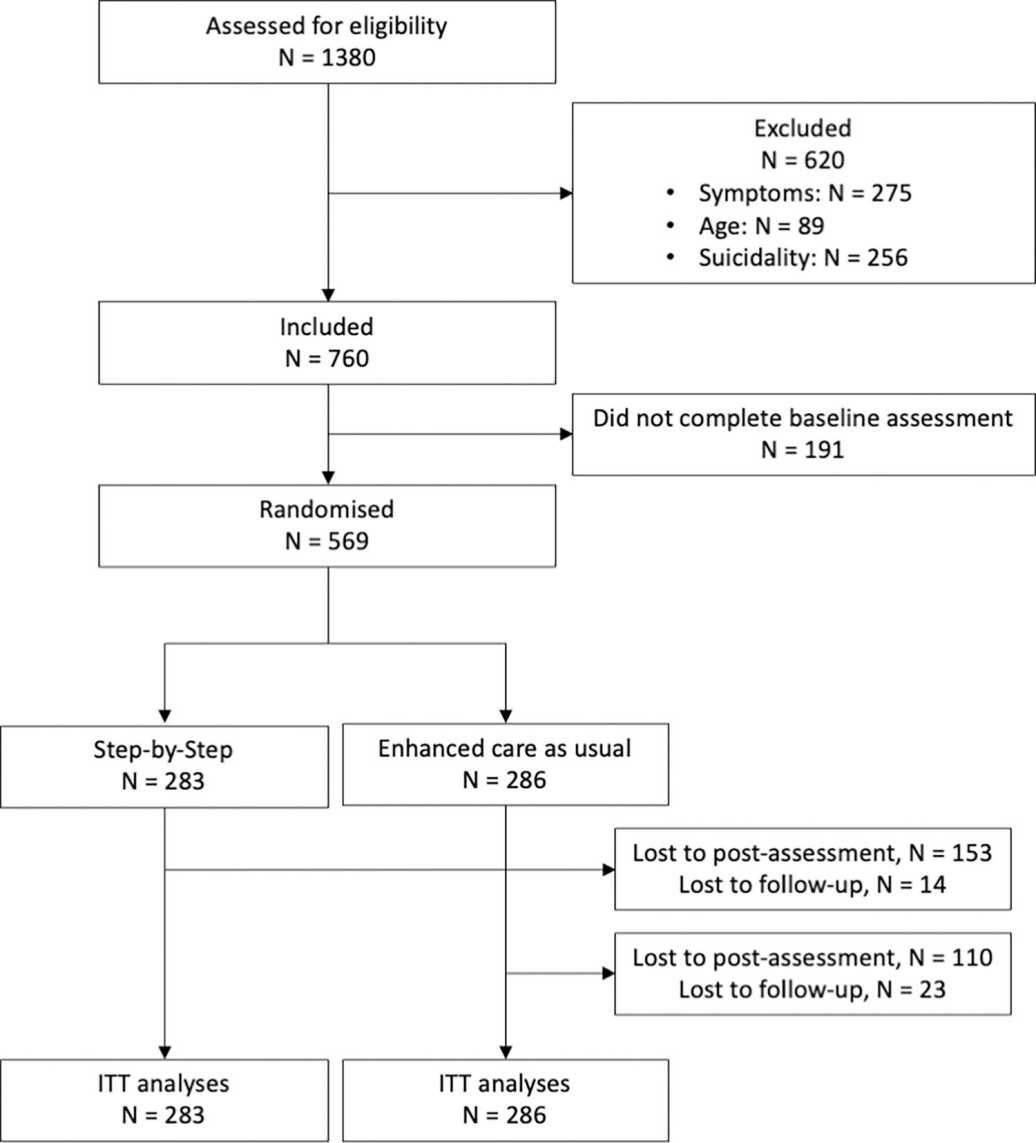
Flowchart.

Of 1,380 persons assessed for eligibility, 569 met inclusion criteria and were randomized (Fig 1). A total of 283 participants were randomized to the intervention and 286 to ECAU. The posttreatment assessment was returned by 53.8% and the 3 months follow-up assessment by 47.3% of respondents (i.e., 46.2% and 52.7% dropout, respectively). The recruitment started on December 9, 2019 and ended on July 9, 2020. The original plan was to start recruitment in November 2019; however, due to the eruption of protests in Lebanon and political unrest in 2019, it was postponed for 1 month. The completion of data collection was also delayed for about 2 months. No other deviations from the protocol occurred.

The sociodemographic characteristics of the participants are summarized in Table 1. The average age was 31.5. The majority of the participants were female (58.3%), and the majority were married (70.1%). Most had primary (32.0%) or secondary education (36.7%), and the majority were unemployed (58.7%). There were no significant differences between intervention and control group. Due to the recruitment procedures, no baseline information was missing for participants.

**Table 1:** Demographic and baseline characteristics^a^.

	Intervention (*n* = 283)	Control (*n* = 286)	Total (*n* = 569)
Age, M (SD)	31.4 (8.5)	31.6 (8.9)	31.5 (8.7)
Female gender	174 (61.7)	158 (55.2)	332 (58.3)
Marital status			
Never married	57 (20.1)	60 (21.0)	117 (20.6)
Married	200 (70.7)	203 (71)	403 (70.1)
Other	26 (9.2)	23 (8.0)	49 (8.6)
Education			
Primary/elementary	91 (32.2)	91 (31.8)	182 (32.0)
Secondary	101 (35.7)	108 (37.8)	209 (36.7)
Undergraduate/BSc	66 (23.3)	66 (23.1)	132 (23.2)
Graduate/MSc	8 (2.8)	9 (3.1)	17 (3.0)
Other	17 (6.0)	12 (4.2)	29 (5.1)
Employment status			
Paid work	21 (7.4)	36 (12.6)	57 (10.0)
Nonpaid work	80 (28.3)	71 (24.8)	151 (26.5)
Student	9 (3.2)	13 (4.5)	22 (3.9)
Unemployed	169 (59.7)	165 (57.7)	334 (58.7)
Other	4 (1.4)	1 (0.3)	5 (1.0)

^a^All cells indicate *n* (%), unless otherwise indicated.

Bsc, bachelor degree; M, means; Msc, master’s degree; SD, standard deviation.

### Primary outcome

The ITT analyses showed significant treatment effects for both primary outcomes, depression (b = −2.81; SE = 0.66; *p* < 0.001), and functional impairment (b = −3.27; SE = 0.98; *p* = 0.001). Effect sizes were moderate for depression (g = 0.48; 95% CI: 0.26; 0.70) and functional impairment (g = 0.35; 95% CI: 0.14; 0.56) (Tables 2 and 3).

**Table 2:** Inferential statistics of treatment outcome measures for complete cases: means, standard deviation, and *N*.

	Baseline		Posttest		3 months follow-up	
	Intervention	Control	Intervention	Control	Intervention	Control
*N*	614	635	238	321	202	272
Primary						
PHQ-9	16.39 (4.15)	16.37 (4.24)	10.19 (6.32)	13.57 (5.48)	9.22 (6.07)	12.54 (5.79)
WHODAS	33.00 (8.66)	33.06 (8.63)	26.62 (9.24)	29.98 (9.34)	25.69 (9.30)	28.76 (9.55)
Secondary						
WHO-5	7.02 (4.26)	7.21 (4.49)	11.02 (5.75)	8.60 (5.26)	11.98 (5.78)	9.04 (5.34)
GAD-7	15.56 (4.81)	15.84 (4.71)	10.24 (5.77)	13.79 (5.52)	9.89 (5.75)	12.77 (5.81)
PCL-5	19.30 (6.38)	19.72 (6.30)	13.84 (7.71)	17.35 (7.08)	12.96 (7.85)	16.20 (7.74)
PSYCLOPS	16.63 (3.70)	16.97 (3.44)	11.57 (5.47)	14.44 (4.62)	10.5 (5.73)	13.07 (5.52)

GAD-7, General Anxiety Disorder-7; PCL-5, PTSD Checklist for DSM-5; PHQ-9, Patient Health Questionnaire; PSYCHLOPS, Psychological Outcomes Profile; WHO-5, WHO Wellbeing Index; WHODAS, WHO Disability Assessment Schedule-12.

**Table 3:** Step-by-step treatment effect estimates.

	Posttest					Follow-up				
	Model 1 MI			Model 2 RFLB		Model 1 MI			Model 2 RFLB	
	B MI (SE)	*p*	Effect size (95% CI)	B Lower bound (SE)	B Upper bound (SE)	B MI (SE)	*p*	Effect size (95% CI)	B Lower bound (SE)	B Upper bound (SE)
Primary										
• PHQ-9	−2.81 (0.66)	<0.001	0.48 (0.26; 0.70)	−4.78 (0.84)	−0.89 (0.83)	−3.63 (0.72)	<0.001	0.61 (0.37; 0.85)	−5.85 (0.99)	−1.27 (0.94)
• WHODAS	−3.27 (0.98)	<0.001	0.35 (0.14; 0.56)	−6.63 (1.37)	0.11 (1.35)	−4.28 (1.13)	<0.001	0.45 (0.22; 0.68)	−8.00 (1.54)	−0.20 (1.41)
Secondary										
• WHO-5	2.57 (0.58)	<0.001	0.47 (0.26; 0.68)	0.90 (0.84)	4.86 (0.80)	2.80 (0.65)	<0.001	0.51 (0.28; 0.74)	1.05 (0.93)	5.66 (0.89)
• GAD-7	−2.56 (0.61)	<0.001	0.46 (0.24; 0.68)	−5.17 (0.83)	−1.06 (0.85)	−2.37 (0.71)	0.001	0.41 (0.17; 0.65)	−5.18 (0.97)	−0.55 (0.88)
• PCL-5	−2.61 (0.75)	<0.001	0.36 (0.16; 0.56)	−6.29 (1.09)	−0.36 (1.16)	−3.00 (0.94)	0.002	0.39 (0.15; 0.63)	−6.54 (1.26)	−0.42 (1.26)
• PSYCLOPS	−2.43 (0.53)	<0.001	0.49 (0.28; 0.70)	−5.04 (0.72)	−1.23 (0.81)	−2.24 (0.74)	0.003	0.40 (0.14; 0.66)	−4.92 (0.89)	−0.74 (0.93)

^a^Treatment effects in model 1 are derived through multiple (150) imputations, assuming missing at random, applying multivariate normal regression, imputation by experimental group, and accommodating arbitrary missing outcome value patterns using an iterative MCMC method. Prescore and background controls (trial dummy, gender, and a dummy for whether a participant had missing values on 1 or more of these characteristics) are included for improved precision of the regression point estimates reported in model 1. Effect sizes reported in model 1 are Hedges g, combining MI estimation results using Rubin’s rules. Treatment effects in model 2 are derived through RFLB procedure, assuming missing not at random for the differential attrition between treatment arms.

B, B coefficient; CI, confidence interval; GAD-7, General Anxiety Disorder-7; MI, according to multiple imputation procedure; PCL-5, PTSD Checklist for DSM-5; PHQ-9, Patient Health Questionnaire-9; PSYCHLOPS, Psychological Outcomes Profile; RFLB, according to Random Forest Lee Bounds procedure; SE, standard error; WHO-5, WHO Wellbeing Index; WHODAS, WHO Disability Assessment Schedule-12.

At 3 months follow-up, the intervention effect was maintained for depression (b = −3.63; SE = 0.72; *p* < 0.001) with a moderate to large effect size (g = 0.61; 95% CI: 0.37; 0.85), and a moderate effect size for functional impairment (b = −4.28; SE = 1.13, *p* < 0.001; g = 0.45; 95% CI: 0.22; 0.68). Estimating worst- and best-case scenarios for the potential impact of selective differential attrition yielded RFLBs for never-attriters (model 2 in Table 3) that are always in the same direction as the ITT results, but with worst-case bounds not statistically significant.

### Secondary outcomes

According to ITT analyses, all secondary outcomes indicated significant results (all *p*-values < 0.002) with moderate SMDs: 0.47 for subjective well-being (WHO-5), 0.46 for anxiety (GAD-7), 0.36 for post-traumatic stress (PCL-5), and 0.49 for personal problems (PSYCHLOPS) (Tables 2 and 3). At 3 months follow-up, the intervention continued to be significantly more effective than ECAU for all outcomes (all *p*’s < 0.03; Table 3), with effect sizes that were comparable to those at posttest.

### Response and complete remission

For the ITT sample, approximately 2 in 5 treated people showed a treatment response (37.1%), versus 1 in 7 (13.3%) in the control group (odds ratio [OR] = 3.8; 95% CI: 2.5; 5.8; *p* < 0.001). Moreover, approximately 1 in 5 (20.8%) treated people completely remitted versus 1 in 20 (4.9%) in the control group (OR = 5.1; 2.8; 9.4; *p* < 0.001). The NNT were 4 (95% CI: 3.3; 5.9) for response and 6 (95% CI: 4.7; 9.4) for complete remission for the ITT sample. The analysis of the sample of participants who experienced complete remission resulted in comparable outcomes to the ITT sample (Table 4).

**Table 4:** Response and complete remission rates^a^.

	ITT				Completers			
	Treatment	Control	OR (95% CI)	NNT (95% CI)	Treatment	Control	OR (95% CI)	NNT (95% CI)
Response	37.1% (105/283)	13.3% (38/286)	3.8 (2.5; 5.8) ^b)^	4 (3.3; 5.9)	40.1% (57/142)	13.3% (24/180)	4.4 (2.5; 7.5) ^b^^)^	4 (2.8; 5.8)
Complete remission	20.8% (59/283)	4.9% (14/286)	5.1 (2.8; 9.4) ^b)^	6 (4.7; 9.4)	21.1% (30/142)	3.9% (7/180)	6.6 (2.8; 15.6) ^b^^)^	6 (4.1; 10.0)

^a)^Response was defined as a 50% reduction in depressive symptoms on the PHQ-9 from baseline to posttest; complete remission was defined as a score of <5 on the PHQ-9.

^b^The associated p-value was *p* < 0.001.

ITT, intention-to-treat analyses; NNT, numbers needed to treat; OR, odds ratio; PHQ-9, Patient Health Questionnaire.

### Other outcomes

Of 283 participants in the intervention condition, 203 (71.7%) finished the introduction session, 119 (42.0%) finished at least 3 of 5 sessions, and 91 (32.2%) finished all sessions. Of the 131 participants who completed the postassessment, approximately half indicated that most or almost all of their needs were met (48.1%). A large majority responded that they were mostly or very much satisfied (89.3%) and that they would come back to the program if they were to seek help again (96.9%). Fidelity checks revealed 6% minor deviations from the treatment protocol, such as a helper not reviewing the story with a user or not reviewing practice exercises. During the trial, one serious adverse event occurred (a hospitalization in the intervention group, unrelated to the intervention).

A qualitative process evaluation was conducted and interviews were held with participants. Feedback on the content, delivery model of the intervention, and dropout was solicited and will be published in a separate paper.

## Discussion

### Summary of the main findings

This study supports the value of digital self-help, in a setting where displaced people were subjected to a range of co-occurring adversities. The fact that the study was able to rapidly complete recruitment during the COVID-19 pandemic shows the value of digital health at a time when physical distancing is required. We found that this new WHO digital mental health intervention, Step-by-Step, is effective in reducing mental health problems among displaced, war-affected Syrians living in Lebanon. Moderate effects on our primary outcome variables, depression and impaired functioning, were found at posttreatment and 3 months follow-up. The intervention also had significant effects on anxiety, post-traumatic stress, well-being, and personal problems, and these effects were maintained at follow-up. With NNTs of 4 (response) to 6 (complete remission) in the ITT sample, the clinical relevance of this intervention is considerable.

### What this study adds

These results are consistent with previous findings showing that e-health in general [38], as well as mobile health apps [13], can effectively reduce mental health problems. However, most of the previous research has been conducted in high-income countries. This study shows that mobile interventions can also be effective in people with mental health problems in a lower-resourced country, among displaced people. The study was conducted in the setting where it should ultimately be implemented, and we think therefore that the results can be generalized well to routine care for displaced persons in Lebanon, as well as to other comparable displaced populations. This can be seen as a strong indication that low cost digital interventions, like Step-by-Step may be used to develop a basic infrastructure for mental health care for common mental disorders in these populations.

### Strengths and limitations

Strengths of this study include the implementation in a country exposed to multiple crises, adaptation to the local context, the large sample size, automated randomization shielding against researcher bias, automated assessment ensuring blinded data collection, the overall strict design and analyses, and the central involvement of Lebanon’s National Mental Health Program, positioning it to scale up this intervention nationally.

Limitations include the high level of dropout, which is inherent to e-mental health interventions and research [39], and for which we had powered the study. Also, we did not conduct clinical diagnostic interviews. Furthermore, we only examined the effects at 3 months follow-up, though effects were maintained at least over that period. Finally, the intervention itself is limited, because it requires digital access, which is inequitably distributed in populations.

### Implications and next steps

Although the results of this study are very encouraging, more research is needed to establish the long-term effects of the intervention. It would also be useful to examine in more depth what happens with those who dropped out of the intervention and what is needed to retain them in the intervention or develop an alternative treatment for them. Because this intervention was specifically aimed at depression, it would also be useful to examine the possibility to develop comparable interventions for other common mental disorders, such as post-traumatic stress disorder and anxiety disorders, especially in displaced populations.

We do think that the results of this study indicate that dissemination of this intervention is justified in Lebanon, as it was tested in exactly the same circumstances where it is to be disseminated. Testing and use in other comparable settings in low- and middle income countries, as well as in displaced populations, should also be considered.

## Conclusions

Despite these limitations, we conclude that the digital intervention had a statistically significant and meaningful effect on depression, functional impairment, anxiety, post-traumatic stress, subjective well-being, and self-identified problems among displaced, war-affected Syrians living in Lebanon. To the best of our knowledge, this study is the first to show that a digital intervention supported by human helpers can contribute considerably to improving mental health among displaced people in a low-resourced setting. Informed by these results and those of a second trial, WHO will release Step-by-Step as an open-access intervention to fill the gap of publicly available evidence-based psychological interventions in global mental health [40]. Lebanon’s National Mental Health Program is now scaling up the intervention in the country.

## Supporting information

S1 Checklist CONSORT Checklist. CONSORT, Consolidated Standards of Reporting Trials.
(DOC)

S1 Protocol Study protocol. (DOCX)

S1 Appendix Details about the statistical procedures. (DOCX)

## Abbreviations

CONSORT: Consolidated Standards of Reporting Trials
COVID-19: Coronavirus Disease 2019
CSQ: Client Satisfaction Questionnaire
ECAU: enhanced care as usual
ITT: intention-to-treat
NNT: numbers needed to treat
OR: odds ratio
PHQ-9: Patient Health Questionnaire
PSYCHLOPS: Psychological Outcomes Profile
RCT: randomised controlled trial
RLFB: Random Forest Lee bound
SMD: standardized mean difference
WHODAS: WHO Disability Assessment Schedule-12
