# Correction: Effects of a WHO-guided digital health intervention for depression in Syrian refugees in Lebanon: A randomized controlled trial

**DOI:** 10.1371/journal.pmed.1004231

**Published:** 2023-04-14

**Authors:** Pim Cuijpers, Eva Heim, Jinane Abi Ramia, Sebastian Burchert, Kenneth Carswell, Ilja Cornelisz, Christine Knaevelsrud, Philip Noun, Chris van Klaveren, Edith van’t Hof, Edwina Zoghbi, Mark van Ommeren, Rabih El Chammay

In Table 2, the numerical values are incorrectly derived from the current clinical trial as well as a sister trial. Please see the correct Table 2 here.

**Table 2:** Inferential statistics of treatment outcome measures for complete cases: Means, standard deviation and N.

	Baseline		Post-test		3 months follow-up	
	Intervention	Control	Intervention	Control	Intervention	Control
N	283	286	142	180	121	159
Primary						
PHQ-9	16.30 (4.19)	16.48 (4.41)	10.76 (6.42)	13.57 (5.47)	9.29 (6.41)	12.86 (5.76)
WHODAS	34.26 (8.76)	34.69 (9.04)	28.12 (9.40)	31.52 (9.28)	26.02 (9.33)	30.27 (9.48)
Secondary						
WHO-5	7.64 (4.49)	7.44 (4.67)	11.43 (5.74)	8.68 (5.11)	12.43 (5.95)	9.19 (5.43)
GAD-7	15.13 (4.90)	15.74 (4.80)	10.36 (5.89)	13.40 (5.41)	9.83 (6.03)	12.69 (5.72)
PCL-5	18.62 (6.54)	19.15 (6.30)	13.81 (7.97)	17.01 (7.19)	12.60 (7.83)	16.05 (7.31)
PSYCHLOPS	16.55 (3.90)	17.25 (3.50)	12.10 (5.82)	14.95 (4.55)	11.39 (6.02)	13.88 (5.30)

## References

[1] Cuijpers P, Heim E, Abi Ramia J, Burchert S, Carswell K, Cornelisz I, et al. (2022) Effects of a WHO-guided digital health intervention for depression in Syrian refugees in Lebanon: A randomized controlled trial. PLoS Med 19(6): e1004025. doi: 10.1371/journal.pmed.1004025 35737665 PMC9223343

