# Supplementary material for: Effects of a WHO-guided digital health intervention for depression in Syrian refugees in Lebanon: A randomized controlled trial
Source: PLoS Med. 2022 Jun 23;19(6):e1004025. doi: 10.1371/journal.pmed.1004025 (PMC9223343; doi:10.1371/journal.pmed.1004025)
Supplement: S1 Checklist — CONSORT, Consolidated Standards of Reporting Trials. (DOC) [file pmed.1004025.s001.doc]

CONSORT 2010 checklist of information to include when reporting a randomised trial*

| Section/Topic | Item No | Checklist item | Reported on page No |
| --- | --- | --- | --- |
| Title and abstract | | | |
|  | 1a | Identification as a randomised trial in the title | Title |
|  | 1b | Structured summary of trial design, methods, results, and conclusions (for specific guidance see CONSORT for abstracts) | Abstract section |
| Introduction | | | |
| Background and objectives | 2a | Scientific background and explanation of rationale | Introduction |
|  | 2b | Specific objectives or hypotheses | Introduction |
| Methods | | | |
| Trial design | 3a | Description of trial design (such as parallel, factorial) including allocation ratio | Methods, Design |
|  | 3b | Important changes to methods after trial commencement (such as eligibility criteria), with reasons | Results, subsection participants |
| Participants | 4a | Eligibility criteria for participants | Methods, subsection Procedures |
|  | 4b | Settings and locations where the data were collected | Methods, subsection Procedures |
| Interventions | 5 | The interventions for each group with sufficient details to allow replication, including how and when they were actually administered | Methods, subsection Study arms |
| Outcomes | 6a | Completely defined pre-specified primary and secondary outcome measures, including how and when they were assessed | Methods, subsection Outcomes |
|  | 6b | Any changes to trial outcomes after the trial commenced, with reasons | Results, subsection participants |
| Sample size | 7a | How sample size was determined | Methods, subsection Analyses |
|  | 7b | When applicable, explanation of any interim analyses and stopping guidelines | Not applicable |
| Randomisation: |  |  |  |
| Sequence generation | 8a | Method used to generate the random allocation sequence | Methods, subsection Procedures |
|  | 8b | Type of randomisation; details of any restriction (such as blocking and block size) | Methods, subsection Procedures |
| Allocation concealment mechanism | 9 | Mechanism used to implement the random allocation sequence (such as sequentially numbered containers), describing any steps taken to conceal the sequence until interventions were assigned | Methods, subsection Procedures |
| Implementation | 10 | Who generated the random allocation sequence, who enrolled participants, and who assigned participants to interventions | Methods, subsection subsection |
| Blinding | 11a | If done, who was blinded after assignment to interventions (for example, participants, care providers, those assessing outcomes) and how | Methods, subsection Procedures |
|  | 11b | If relevant, description of the similarity of interventions | Methods, subsection Study arms |
| Statistical methods | 12a | Statistical methods used to compare groups for primary and secondary outcomes | Methods, subsection Analyses |
|  | 12b | Methods for additional analyses, such as subgroup analyses and adjusted analyses | Methods, subsection Analyses |
| Results | | | |
| Participant flow (a diagram is strongly recommended) | 13a | For each group, the numbers of participants who were randomly assigned, received intended treatment, and were analysed for the primary outcome | Results, subsection Participants, Figure 1 |
|  | 13b | For each group, losses and exclusions after randomisation, together with reasons | Results, subsection Participants, Figure 1 |
| Recruitment | 14a | Dates defining the periods of recruitment and follow-up | Results, subsection Participants |
|  | 14b | Why the trial ended or was stopped | Results, subsection Participants |
| Baseline data | 15 | A table showing baseline demographic and clinical characteristics for each group | Table 1 |
| Numbers analysed | 16 | For each group, number of participants (denominator) included in each analysis and whether the analysis was by original assigned groups | Figure 1, flowchart |
| Outcomes and estimation | 17a | For each primary and secondary outcome, results for each group, and the estimated effect size and its precision (such as 95% confidence interval) | Tables 2 and 3 |
|  | 17b | For binary outcomes, presentation of both absolute and relative effect sizes is recommended | Table 4 |
| Ancillary analyses | 18 | Results of any other analyses performed, including subgroup analyses and adjusted analyses, distinguishing pre-specified from exploratory | Results, Subsection Other outcomes |
| Harms | 19 | All important harms or unintended effects in each group (for specific guidance see CONSORT for harms) | Results, Subsection Other outcomes |
| Discussion | | | |
| Limitations | 20 | Trial limitations, addressing sources of potential bias, imprecision, and, if relevant, multiplicity of analyses | Discussion, Subsection Strengths and limitations |
| Generalisability | 21 | Generalisability (external validity, applicability) of the trial findings | Discussion, Subsection What this study adds |
| Interpretation | 22 | Interpretation consistent with results, balancing benefits and harms, and considering other relevant evidence | Discussion, Subsection Summary of the main findings |
| Other information | | |  |
| Registration | 23 | Registration number and name of trial registry | Last sentence of Abstract; and at the end of the text |
| Protocol | 24 | Where the full trial protocol can be accessed, if available | Reference 16 |
| Funding | 25 | Sources of funding and other support (such as supply of drugs), role of funders | Funding section |

*We strongly recommend reading this statement in conjunction with the CONSORT 2010 Explanation and Elaboration for important clarifications on all the items. If relevant, we also recommend reading CONSORT extensions for cluster randomised trials, non-inferiority and equivalence trials, non-pharmacological treatments, herbal interventions, and pragmatic trials. Additional extensions are forthcoming: for those and for up to date references relevant to this checklist, see [www.consort-statement.org](http://www.consort-statement.org/).
