# Supplementary material for: Effects of a WHO-guided digital health intervention for depression in Syrian refugees in Lebanon: A randomized controlled trial
Source: PLoS Med. 2022 Jun 23;19(6):e1004025. doi: 10.1371/journal.pmed.1004025 (PMC9223343; doi:10.1371/journal.pmed.1004025)
Supplement: S1 Protocol — (DOCX) [file pmed.1004025.s002.docx]

**Increasing Access to Mental Health Care for People Living in Lebanon: an E-Mental Health Intervention**

**Public title: “Testing e-Mental Health in Lebanon”**

Study protocol for review by WHO Ethical Review Committee.

Version 7

NOTE: tracked changes in this document reflect proposed changes to the document after review of Version 6 by the ERC.

Contact person:

Mark van Ommeren, PhD

World Health Organization (WHO)

Department of Mental Health and Substance Abuse

Email: [vanommerenm@who.int](mailto:vanommerenm@who.int)

Telephone: +41-22-791-3619

**PROJECT SUMMARY**

***Background:***

Mental health problems affect one in five people globally, with depression reported to be the leading cause of global burden of disease (Ferrari et al 2013). There is a need for evidence-based psychological interventions that can be used in settings which are resource pressured. Recently updated World Health Organization (WHO) guidelines for the treatment of depression in adults, recommend behavioural activation noting that different treatment formats for consideration include (a) individual and/or group face-to-face psychological treatments delivered by professionals and supervised lay therapists, as well as (b) self-help psychological treatment. However, evidence from low- and middle-income countries on such self-help interventions is scarce (Arjadi et al 2015). WHO aims to test the feasibility, acceptability, effectiveness and cost-effectiveness of an e-mental health guided self-help intervention based on behavioural activation, delivered through the internet using computers, tablets or smartphones in Lebanon and guided by a non-specialist care provider. An evidence based e-mental health intervention for depression could improve access to help for people in under-resourced areas.

***Objectives:***

The study objective is to determine whether a guided e-mental health intervention called Step-by-Step can be feasibly delivered to people living in Lebanon, and to test the effectiveness and cost-effectiveness of this intervention. This will be achieved by conducting a non-controlled pilot study of the intervention with people living in Lebanon (i.e. Lebanese nationals, Syrian displaced people and other populations resident in Lebanon) who are experiencing depression (*Study Phase 1*), qualitative process evaluations (*Study Phases 2, 4* and *6*), a feasibility and a definitive randomised controlled trial (RCT, *Study Phases 3* and *5*).

***Design and methodology:***

*Study Phase 1* will comprise a non-controlled pilot study of the intervention to investigate the feasibility of delivery, acceptability and safety of the intervention. Measures on functioning, depression and anxiety symptoms, wellbeing and perceived problem magnitude will be completed by approximately 200 participants with symptoms of depression, both before and after use of the intervention.

*Study Phases 2, 4* and *6* will be process evaluations, using qualitative methods including key informant interviews with study participants (including drop outs) and facilitators of the intervention, as well as local key stakeholders, to further understand aspects related to the feasibility and acceptability of the intervention.

In *Study Phase 3,* a feasibility RCT (n= 60) will be conducted among people living in Lebanon where participants will be recruited from primary health care (PHC) centres, similar social care and community centres and using other methods of advertisement (e.g. social media, posters, radio adverts etc.). After informed consent, Syrian displaced people (n= 15) and other Lebanese residents (n= 15) with high levels of psychological distress (i.e. a score of ≥10 on the Patient Health Questionnaire [PHQ-9] and a score of >16 on the WHO Disability Assessment Schedule [WHODAS] 2.0), will receive Step-by-Step, while those in the control arm (n= 15 for each group, respectively) will receive enhanced care as usual (ECAU). Pre- and post-treatment assessments include depression, functional disability, anxiety, wellbeing, posttraumatic stress disorder (PTSD) symptoms, perceived problems for which the person seeks help and user satisfaction with the intervention. A follow-up assessment will be done three months after the post-assessment.

*Study Phase 5* involves a definitive RCT including Syrian displaced people (n= 568) and Lebanese people (n= 568). Participants will be randomly allocated (1:1) to either Step-by-Step or ECAU. The same assessments as in the feasibility RCT will be used.

***Time frame:***

The study will take place over 36 months. *Study Phase 1* and *2* are expected to take approximately 12 months and *Study Phases 3 to 6* are expected to take approximately 24 months.

***Expected outcomes:***

Outcomes from this project include understanding about the feasibility, acceptability, effectiveness and cost-effectiveness of a guided e-mental health intervention in a middle-income country, as well as capacity building of the Lebanese Ministry of Public Health (MoPH) and other project partners. Longer term gains, assuming the intervention is proven effective in RCTs, include the availability of an e-mental health intervention to be disseminated by the MoPH in Lebanon, and a generic version of the intervention suitable for adaptation for other contexts.

**GENERAL INFORMATION**

**Project title:**

Increasing Access to Mental Health Care for People Living in Lebanon: an e-Mental Health Intervention

**Public title:**

Testing e-mental health in Lebanon

**Name and address of the sponsors/funders:**

Elrha – Research for Health in Humanitarian Crises (r2hc)

Save the Children Wales / Achub y Plant Cymru

8 Cathedral Rd, Cardiff CF1 9LJ

UK

Fondation d’Harcourt

Place de Chevelu 6

1201 Geneva

Switzerland

**Name and title of the investigators:**

Mark van Ommeren, PhD

Public mental health advisor

World Health Organization (WHO)

Department of Mental Health and Substance Abuse

Email: [vanommerenm@who.int](mailto:vanommerenm@who.int)

Telephone: +41-22-791-3619

Responsibility: Responsible Officer within WHO for the study

Rabih El Chammay, MD

Head of National Mental Health Programme

Ministry of Public Health, Lebanon

Email: rabihelchammay@gmail.com rchammay@moph.gov.lb

Tel: +961 1 611 174 ext.: 127

Responsibility: Principal investigator (PI)

Pim Cuijpers, Professor

Department of Clinical Psychology

VU Amsterdam

Netherlands

Tel:

Fax:

Email: p.cuijpers@vu.nl

Responsibility: Study design, quantitative data analyses

Eva Heim, PhD

Department of Psychology

University of Zurich

Binzmuehlestrasse 14/17

8050 Zurich

Tel: 0041 44 635 73 26

Email: [eva.heim@psychologie.uzh.ch](mailto:eva.heim@psychologie.uzh.ch)

Responsibility: Trial coordinator

**Name and title of the collaborators:**

Claire Whitney, MIA, LICSW

Regional Mental Health and Psychosocial Support Advisor – Middle East

International Medical Corps

Regional Technical Unit – Middle East

Global Investment House #9, 4th Floor

Abd Alhamid Sharaf Street

Shmeisani

Amman, 11185,

Jordan

Tel: +962 790 858 309

Email: [cwhitney@internationalmedicalcorps.org](mailto:cwhitney@internationalmedicalcorps.org)

Dr Sami Richa (PhD Bioethics)

Head of psychiatry department, Faculty of Medicine, Saint Joseph University

AFMM (Association Francophone pour les Malades Mentaux) affiliated to St Joseph’s University, Medical Faculty

Damascus Road – Riad el Solh

11-5076

Beirut, Lebanon

Tel: +96 (0)13 225 960

E-mail: [sami.richa@usj.edu.lb](mailto:sami.richa@usj.edu.lb)

Christine Knaevelsrud, PhD

Department of Education and Psychology

Division of Clinical Psychological Intervention

Freie Universität Berlin | FUB

Kaiserswerther Str. 16-18

14195 Berlin

E-mail: christine.knaevelsrud@fu-berlin.de

**Name and title of the international advisors**

Andreas Maercker (Prof)

University of Zurich (UZH)

Department of Psychology

Division of Psychopathology & Clinical Intervention

Binzmuehlestr 14/17

CHO8050

Zürich

Switzerland

+41 (0)44 635 7326

Email: e.heim@psychologie.uzh.ch; maercker@psychologie.uzh.ch

**Address and telephone numbers of research site:**

National Mental Health Programme (NMHP)

Ministry of Public Health,

4th Floor Lebanese University Central Directorate,

Museum Square,

9800,

Beirut,

Lebanon

Tel: +961 1 611 174 ext.: 127

**RATIONALE AND BACKGROUND INFORMATION**

**Background**

Mental health problems affect one in five people globally, with depression reported to be the leading cause of global burden of disease (Ferrari et al 2013). The median annual mental health expenditure per capita in low and middle income countries is less than US$0.25 and approximately 85% of people with mental health problems do not have access to the treatment they need (WHO 2012).

There is a need for evidence-based psychological interventions that can be used in settings which are resource pressured. Such interventions should be suitable for scale-up, as well as being appropriate for diverse socio-cultural groups. E-mental health, which is the use of electronic devices as a means to provide mental health interventions, could be a way to increase the coverage of evidence based psychological interventions in a sustainable manner. Such interventions have been found to be as effective as face to face interventions (Andersson et al 2014; Andrews et al 2010) and in addition, have been shown to reduce symptoms of mental disorders in routine care (Andersson and Titov 2014). The evidence for the efficacy of e-mental health is so compelling that e-mental health programmes have been included in a number of countries’ national mental health strategies and treatment guidelines, including the Netherlands, the United Kingdom, Australia, New Zealand, Scandinavian countries (Moock 2014) and more recently, Lebanon (Republic of Lebanon Ministry of Public Health 2015).

The majority of current e-mental health interventions are available through secure websites that are accessed on a personal desktop computer or laptop. However, with the increasing use of mobile devices in recent years there is a trend towards the use of smartphones and tablets as access devices for internet-based services, including mobile health applications (Rai et al., 2013). Mobile health (mHealth) is the use of mobile technologies and mobile apps as a means to provide health interventions. Utilising mHealth has become a promising scaling-up strategy for mental health interventions (Ruzek et al 2016). mHealth apps are usually optimized for use on mobile devices and therefore differ in design und user interface from classical webpages. In regard to mental health, the majority of mHealth apps focuses on unguided and self-directed skill learning and the transfer of health promoting strategies into everyday life.

Recently updated WHO guidelines on the comparative effectiveness of different formats of psychological treatments for depressive disorder in low resource settings recommend behavioural activation (BA), cognitive behavioural therapy (CBT), interpersonal psychotherapy (IPT), or problem-solving treatment with the following comment:

*“…Different treatment formats for consideration include (a) individual and/or group face-to-face psychological treatments delivered by professionals and supervised lay therapists, as well as (b) self-help psychological treatment….While face-to-face psychological treatment or guided self-help psychological treatment are likely to have better outcomes than unguided self-help, the latter may be suitable for those people who either (a) do not have access to face-to-face psychological treatment or guided self-help psychological treatment or (b) are not willing to access such treatments.”* (WHO 2015)

However, evidence from low- and middle-income countries on such self-help interventions is scarce. Evidence from high-income countries showed that the success of e-mental health care can depend on its integration into, and affordability as part of, existing health services (Moock 2014); whether or not it is guided by a coach or care provider (Spek et al 2007; Andersson and Cuijpers 2009); and technological factors, such as internet coverage and electronic device literacy and usage (van’t Hof et al 2011). WHO aims to test the feasibility, acceptability, effectiveness and cost-effectiveness of an e-mental health intervention in Lebanon, a middle income country with limited available mental health resources. Lebanon has a mental health plan that encourages the use of e-mental health as an affordable delivery option for evidence based mental health care (Republic of Lebanon Ministry of Public Health 2015).

According to the International Telecommunications Union (2017), a substantial proportion of the population in Lebanon has access to mobile phones (81%) and the Internet (78%), and this percentage is likely to rise in the coming years. The literacy rate is 88% for adults and 96% for youths. With regard to Syrian refugees, the United Nations High Commissioner for Refugees (UNHCR) reports 100% 3G coverage among refugees in Lebanon, and 68% of mobile ownership among refugees worldwide (UNHCR 2016). These data suggest that many Lebanese and Syrian displaced people have the means to access an e-health intervention.

Lebanon has limited resources and significant need for mental health and psychosocial support services. The country has a history of political turmoil and conflict. Civil war, border tensions, war with neighbouring countries and the more recent conflict in Syria have all affected Lebanon. The UNHCR (2014) reports that the impact of the Syrian crisis - including on the economy, demographics, political instability, and security - continues to deepen across Lebanon. With more than 1.1 million registered refugees and many more undocumented persons of concern, Lebanon's resources are extremely stretched.

A national representative survey (n=2857) conducted in Lebanon before the Syrian civil war showed that one in six people met criteria for at least one mental disorder, with 27.0% of these classified as “serious”. Only one in nine Lebanese respondents with a mental disorder had ever obtained any treatment (Karam et al 2006). It is to be expected that this situation has worsened since 2011, with the recent influx of displaced persons affected by the conflict in Syria. Refugees are at heightened risk of mental disorders, as they have been exposed to multiple war stressors including interpersonal violence, destruction of their livelihoods as well as extreme stress during their flight from their home country (Alpak et al 2015). Affordable and accessible mental health care is very limited in Lebanon. Ministry of Public Health (MoPH) data from 2015 (Republic of Lebanon Ministry of Public Health 2015) reports that there are 1.26 psychiatrists per 100,000 population and 3.42 psychologists per 100,000 population and that 97% of the mental health care workforce work in private practice in Lebanon.

Despite high need, access to mental health services in Lebanon is limited and services are predominantly provided by specialists in the private sector which makes them unaffordable to many. The MoPH has however been working to increase coverage by integrating mental health care into primary health care (PHC) settings. The MoPH has trained staff in more than 60 PHC centres nationwide on the assessment and management of people with mental health conditions following WHO mental health Gap Action Programme (mhGAP) guidelines. Aside from these services, publicly available services are limited.

Mental health care is mostly concentrated in Beirut with the trained PHC facilities providing care in other parts of the country. Hence, access to mental health services is mainly limited to people of high socioeconomic status from urban areas. This inequity has been further heightened since the influx of more than 1 million Syrian refugees which has contributed to further strain on the health system and limited available services. Stigma concerning mental health problems and the lack of funding and for mental health services across Lebanon are further barriers to accessing care. This all contributes to the estimated mental health treatment gap in Lebanon of 90% and highlights the importance of a mental health intervention that can be delivered widely despite the limited human resources, limited funding and stigmatising beliefs that currently reduce access to care.

Few studies have examined the effect of self-help e-mental health interventions outside of high income countries (Arjadi et al 2015), with culturally diverse populations or in conflict or post-conflict settings. One small pilot trial of a higher resource intensity online writing course in Iraq (with considerable guidance from a therapist) showed a substantial effect size (Knaevelsrud et al. 2015).

Before delivering an intervention to a population which linguistically and culturally differs from the original intended beneficiaries, it should be locally adapted (Bernal and Sáez Santiago 2006; Castro et al 2004; Castro et al 2010). Research indicates that culturally adapting evidence-based interventions (which were originally developed for western populations) significantly enhances their effectiveness (Benish et al 2011; Smith et al 2011). Systematic methods of cultural adaptation of psychological measures have been documented (e.g. the method of cognitive interviewing (Van de Vijver and Leung 1997). Step-by-Step has been culturally adapted for Lebanon, and the results of this process have been published in the meantime (Abi Ramia et al., 2019)

**Current Project**

This project is seeking to address the high burden of disease associated with depression symptoms through a potentially cost-effective and scalable evidence-based intervention in Lebanon. We seek to conduct a non-controlled pilot study, a feasibility randomised controlled trial (RCT) and a definitive RCT of a new low intensity, minimally guided e-mental health intervention called Step-by-Step (Burchert et al., 2019; Carswell et al., 2018). There will be a focus throughout on determining whether Step-by-Step is suitable to address the mental health needs of Syrian displaced people and other people living in Lebanon (i.e. Lebanese nationals and other displaced groups such as Palestinians).

**Overall study goal and objectives**

**Goal**: To determine the feasibility, acceptability, effectiveness and cost-effectiveness of Step-by-Step for use among people residing in Lebanon, including Lebanese nationals, Syrian displaced people and other populations resident in Lebanon.

**Specific Objectives:**

- To determine whether Step-by-Step can be feasibly delivered and is acceptable to Lebanese nationals, Syrian displaced people and other populations living in Lebanon (*Study Phase 1).*
- To conduct a non-controlled pilot study of the intervention with people living in Lebanon who are experiencing depression (*Phase 1)*;
- To understand the perceptions of participants, facilitators, and other key stakeholders with regard to Step-by-Step, and to further adapt the intervention to the local context *(Study Phases 2, 4* and *6).*
- To test recruitment and randomisation procedures in a feasibility RCT in order to inform a full-scale, definitive RCT *(Study Phase 3).*
- To test effectiveness and cost-effectiveness of Step-by-Step through a definitive RCT *(Study Phase 5)*.

**The intervention**

Step-by-Step is brief (5-sessions) and has been designed to primarily treat depression symptoms (Carswell et al., 2018). Behavioural activation is the main active therapeutic agent in the intervention, as it is easy for users to engage with and has a very strong evidence base for depression (Cuijpers et al 2007; Ekers et al 2014). Behavioural activation is amenable to a minimally guided, internet delivery model (Ly et al 2014). The intervention comprises of exercises to increase behavioural activation, including pleasurable activities and social support; and additional strategies to support this, including two stress management techniques, a gratitude and a positive self-talk exercise. Given the inclusion of these additional strategies, the effect of the intervention on anxiety symptoms will also be assessed. The intervention will be available online, with some minimal guidance in its use from trained research assistants called e-helpers.

The intervention comprises of a narrative approach as a means of transmitting information and strategies for coping with depression, with tasks for the user to practice over the week. The story and its illustrations are being sensitively adapted to the local context, considering linguistic and cultural nuances within the ethnic populations residing in Lebanon (broadly speaking, Lebanese, Syrian and Palestinian people). Results of the adaptation process have been published by Abi Ramia et al. (2019).

There will be four versions of Step-by-Step, two male and two female. For each gender there will be two different styles of dress, reflecting local culture. The text content will be slightly adapted across these four versions, but the active therapeutic content will stay the same. This accounts for gender differences and provides broad tailoring based on the main cultural groups of the target users. *Study Phase 2* will provide further information on how to adapt Step-by-Step to make it inclusive for different groups, e.g. with regard to age, living conditions, family situation, etc. The language in the online consent form and intervention is simple, in order to engage users who have lower literacy levels, and audio guidance for all components of the intervention (e.g. including assessment questions) will be provided to improve understanding and encourage practice of key elements.

Step-by-Step is supported by trained non-specialist research assistants (called “e-helpers”), who will have weekly phone or message-based contact with users to provide support and guidance lasting around 15 minutes per week. E-helpers will likely have a background in psychology or social care and will work under the regular supervision of trained mental health practitioners (e.g. a clinical psychologist).

This design ensures that the intervention is easy to disseminate and potentially scalable in areas where there is minimal mental health care provision. Such a design has been used before in a range of settings including high income and lower resourced settings in East Asia and the Middle East (e.g. Berger et al 2013, Carlbring et al 2011, Knaevelsrud et al 2015, Kleiboer et al 2015, Wang et al 2013). While this remote model may appear at odds with many common beliefs concerning psychological interventions, systematic reviews have shown compelling evidence supporting such non “face to face” psychological interventions (e.g. Andersson et al 2014).

The content of Step-by-Step was developed with experts in psychological care, e-mental health and global mental health. It has gone through extensive peer-review, with over 30 external experts reviewing the intervention. The development of the generic (English language) version of the intervention and the culturally adapted (Arabic language) version have been completed. The most recent version of the intervention content (the narrative story) for the first female version can be found in Appendix A^[[1]](#footnote-2)^.

## The Lebanese setting

We chose Lebanon as the setting for this study for various reasons. In Lebanon, the MoPH is well placed in being the main project partner. It will coordinate its already strong synergies with leading scientists at St Joseph’s University, with International Medical Corps (IMC) and other key players in mental health care provision in Lebanon to maintain this as a multi-stakeholder initiative. The WHO Country Office and Regional Office have provided their support for this project. This collaboration of public health, research and field-based health care provision organisations will build on existing strong partnerships and provide the necessary knowledge and skills for intervention adaptation, piloting the delivery model, as well as implementing the RCTs and the process evaluations.

The treatment package will be offered to adults in Lebanon with symptoms of depression. The delivery model and recruitment strategies are described separately for each study phase (see below). As explained in our letter to the WHO ERC in May 2017 (Annex M), the recruitment strategy was changed from a primary focus on PHC facilities to including the wider community in Beirut.

Some information on the Lebanese setting was already provided above (see Background). Mental health care services are complex in Lebanon, and services are spread in a fragmented way over a number of public and private providers, with inequality in distribution and access to health care facilities. The MoPH is currently making efforts to deliver WHO mhGAP training and resources for mental health care through these PHC facilities. They have successfully trained staff in more than 60 of these PHC facilities, meaning that these centres have functioning specialist supervision and referral networks for mental health care. The NGO and international NGO (INGO) partners support the mental health care provision in many of these public PHC facilities by offering integrated multidisciplinary care. Private primary care services are also an important means of access to health care in Lebanon and often have established referral networks to mental health practitioners. Step-by-Step will be piloted as the first step in a stepped care model for both public and private settings and also, in order to extend care to the wider community, participants will be recruited by such methods as flyers, posters as well as social media, television and newspaper ads.

**Delivery of the intervention in Lebanon**

The delivery model – an e-mental health intervention with remote support from e-helpers – has been developed to address problems with Lebanon’s limited mental health workforce, its low coverage of specialist services and the stigma associated with attending mental health services. The MoPH Lebanon and partners envision this e-mental health intervention as a first step in a stepped care model to ease the pressure on this already overburdened system. The intervention has been designed to reduce the amount of time health care workers spend with clients each week and reduce the number of referrals to specialists for psychological interventions. If the intervention is unsuccessful, users can proceed to the next step of care (e.g. a face to face intervention).

Step-by-Step is delivered through (a) users’ own smartphones (via an iOS/Android app) or via a website (can be used on a computer or smartphone) or (b) tablets placed in health/community facilities. Though internet connectivity and mobile ownership are relatively widespread in Lebanon, the public (MoPH-supported) network of PHC facilities tend to serve groups of lower socio-economic status who may not be able to afford access to internet or a native app.

To offer equal opportunity of access to care, in *Study Phase 1* we provided four PHC facilities with tablets for participants to use in a private room in the facility. These tablets were not used. However, other PHC facilities and NGOs working in the mental health sector reported about people being interested in the intervention but having no access to a device or internet. Use of tablets will be a special topic for the process evaluation in *Study Phase 2,* and tablets will be provided to centres upon request in *Study Phases 3* and *5.* Stigma around mental health is a barrier to mental health seeking behaviour in Lebanon, so to avoid the use of these tablets becoming stigmatised, the tablets will also have some simple health promotion materials available for general use.

The delivery model was first planned to be based on PHC facilities only. As described in our letter to the ERC in May 2017 (Annex M), we decided to test an alternative access model in the wider community (e.g. through social media ads) and in private health facilities where users can access the intervention using their own device and have connection to the internet. The plan for a model which included face to face recruitment and support of participants by health workers and/or research assistants was replaced with a model of remote, online registration and support for three important reasons. Firstly, the location of the clinics for recruitment, the predicted number of participants that could be recruited from each clinic, and the number of staff required, suggested the original model would not be feasible in the previously planned setting and therefore would not meet the WHO and Lebanese Ministry of Public Health objectives for the project. Secondly, as advised by world-leading e-health experts (who attended one of our consultations in Lebanon) we learned that a model whereby individuals are supported remotely (e.g. by telephone or messaging) instead of face to face was feasible and would be more sustainable. Such models are often used in mental health services in a range of countries (e.g. This Way Up in Australia). Thirdly, we wanted to expand the research to focus on a wider range of participants and to include individuals who are unlikely to attend government supported PHCs. To do this, a remote model is suitable. We are of the opinion that this new model strengthens both the intervention and the research in general.

If needed, participants can use wireless Internet access in PHC facilities to download the Step-by-Step app for further use at home. Independent of the device used, the content will look like a comic book embedded within a website platform. The user area will be password protected and will automatically log users out after a number of minutes of inactivity. Stigmatising or medicalising words associated with mental disorder will not be used in the intervention.

**Study design**

As in our previous studies on low intensity psychological interventions, our research strategy is informed by the *UK Medical Research Council Framework for the Development of Complex Interventions* (Craig et al 2008), which recognizes iterations of: a) Intervention Development; b) Feasibility and Piloting; c) Evaluation; and d) Implementation. This key framework for development of interventions recommends exploratory non-controlled pilot studies and feasibility RCTs prior to large scale RCTs in order to address uncertainties such as problems of acceptability, compliance, delivery of the intervention, recruitment and retention. Accordingly, the planned study will encompass the following six phases:

*Study Phase 1* will comprise a non-controlled pilot study of Step-by-Step that will inform us about the feasibility of delivery, acceptability and safety of the intervention. At this stage, Step-by-Step will be delivered online via a website only. Participants in this phase will be individuals with symptoms of depressive disorder (see below) and will be recruited through adverts in community and PHC settings, social media ads, flyers and newspapers. The pilot study will also assist with identifying issues around training, supervision and appropriate outcome measures. Quantitative data will be collected on variables such as: symptoms of depression and anxiety, functioning, and well-being both before and after the intervention; participant drop-out rates, number of sessions attended by participants, and treatment fidelity.

*Study Phases 2, 4 and 6* will be process evaluations, using qualitative methods including key informant interviews with participants (including drop outs) and facilitators of the intervention, as well as local key stakeholders. Interviews will explore, among other aspects related to the intervention, barriers and facilitators to treatment engagement and adherence, and will seek suggestions for improvement. Feasibility will be assessed on the basis of quantitative data (such as drop out rates, number of people registering and completing the intervention), qualitative data (e.g. perceptions of clients and staff concerning whether the intervention can be delivered) and a retrospective assessment of strengths, weaknesses and problems with the delivery model. Acceptability will be determined by the drop-out rates (in comparison with similar existing interventions) and additional qualitative information. Safety will be determined through a consideration of any (unlikely) adverse events that may occur during the study.

In *Study Phase 3,* we will conduct a feasibility RCT that will further inform us about the feasibility, safety and delivery of the intervention*.* At this stage, Step-by-Step will be accessible either via an iOS/Android app, or via a website. This RCT, which is not powered to show efficacy, will provide the necessary information to inform the definitive RCT methods (e.g. drop-out rates, recruitment strategies, etc.).

In *Study Phase 5*, we will conduct a fully powered definitive RCT to evaluate effectiveness and cost-effectiveness of Step-by-Step.

**Study Methodology**

**Study Phase 1: Exploratory (non-controlled) pilot study to evaluate the feasibility and acceptability of an online-version (website) of Step-by-Step in Lebanon**

**Purpose**

To conduct a pilot study of the intervention with the target populations. This phase can be broken down into the following steps:

1. Preparatory work for the feasibility pilot;
2. Participant recruitment including self-screening;
3. Testing the intervention;

**Overview**

*Study Phase one* is a pilot study with no control group that is intended to inform us about the feasibility of delivery, acceptability and safety of the intervention in Lebanon. It will identify issues around helper supervision, participant recruitment, retention, utility and appropriateness of outcome measures and aspects of the e-helper model, to inform a further RCT. Programmers will programme an online version of the intervention in relevant Arabic dialect and finalise study materials. Applicants with symptoms of depressive disorder will be eligible to participate in the pilot testing of the intervention.

The intervention is aimed at any adults living in Lebanon and experiencing symptoms of depression. It was primarily designed for Lebanese, Syrian and Palestinian people living in communities in Lebanon affected by adversity, most likely of low socio-economic status, and visiting government funded PHCs. It is also relevant to those of higher socio-economic status and will therefore be tested in multiple populations. An online intervention was deemed suitable for all Lebanese residents, as anecdotal reports suggest smartphone usage and internet connectivity is widespread, even among refugees. UNESCO reports that Lebanon has a literacy rate of 90% (though literacy rates are not recorded for displaced populations).

In addition to this lower SES group, the intervention will be available to higher SES individuals through recruitment from private family medicine centres and the community (e.g. flyers, adverts) or possibly online advertising. Including these populations in the study is important as it will provide more detailed information on which groups can feasibly use the online intervention.

**Phase 1- part a: Preparation for the pilot**

The research team has been working on producing preparatory materials for the pilot, including the outline of a training manual and Standard Operation Procedures (SOPs) (appendix B and C). The model includes e-helpers providing support to participants throughout the use of the intervention. E-helpers will likely have an academic background in psychology, social work, occupational therapy, nursing or other such fields relevant to mental health care and will be hired, trained and supervised to provide support and adhere to research protocols. Knowledge and therapeutic skills to be covered will include: working with people with depression and other mental health difficulties, identifying and dealing with crisis situations and responding to adverse events. Training will also cover the intervention itself, including how e-helpers will guide users and use the online system.

**Phase 1- part b: Participant recruitment and self-screening**

**Study setting and entry criteria**

The study will be conducted through public and private health facilities and from additional community settings, (e.g. community centres). Because attrition rates in e-mental health studies can be high (van Ballegooijen et al., 2014), we aim to recruit approximately 200 adults with the expectation that approximately one third will drop out before the end of the intervention. We aim for a mixture of male and female adults. Interested people will learn about the intervention by seeing the posters, flyers or other recruitment materials in community settings or at a health facility. We have attached content of an example flyer (appendix D).

In all recruitment settings, posters will be displayed throughout the recruitment period. Interested people will be free to access the self-screening online, study information and informed consent procedures (see appendix E). In four of the government supported PHCs, participants will be informed that they have the choice to use the onsite tablet or their own device. All information assessments and the intervention will be provided online in Arabic, and possibly English should the need arise, with remote support from e-helpers where necessary.

Inclusion criteria will be any adult person living in Lebanon who:

- is experiencing depression based on scoring 10 or above (the clinical cut-off) on the PHQ-9;
- has access to a device for intervention delivery or is willing to use one in the health centre;
- is an adult (18 and older)

Exclusion criteria will be:

- People who have plans to end their life
- Minors (under 18 years of age)

With regards to health centre recruitment, no recruitment will occur from specialist care facilities. We envisage there will be at least five participating primary health care facilities, with additional private non-specialist centres (e.g. family medicine department at a hospital in Beirut). Clinicians will not be directly involved in the research nor the recruitment of participants. They may direct a potential participant to the materials (e.g. poster or tablet) if they believe this may compliment the routine care provided to the person, but to ensure there is no coercion to participate, all information on the study will be provided online or by e-helpers through messages or telephone calls. Applicants will apply to join the study by navigating to a web page themselves and following on screen instructions.

On the website, they will complete an initial self-screening measure PHQ-9 (Kroenke et al 2001), and indicate their age. If an individual meets the inclusion criteria, they will be able to register for further use of the site and complete informed consent (see appendix E), demographics and their baseline assessment. Applicants will be reminded that they are free to decline to participate or withdraw at any time without affecting their routine care. Applicants will also be asked to confirm their contact preferences for regular support from e-helpers (e.g. contact from e-helpers by telephone, online messaging system, or not at all). Applicants will be able to contact e-helpers throughout the self-screening and recruitment process for free using telephone or messaging services. If the individual does not score over the clinical cut off on the PHQ-9, a box will appear saying that the intervention may not be a good fit for them and to discuss their support needs with a health care worker should they wish.

If the participant answers 2 or above on item 9 of the PHQ-9 and/or affirmatively to a question in appendix F, an on-screen message will appear explaining that they may need additional mental health support (along with information on sources of assistance) with tips for self-care. They will not be able to participate in the research. Please see pages 29 and 30 for more details on risk of suicide and self-harm.

**Phase 1, part c: Testing the intervention**

After completing consent procedures (appendix E), the participant will complete additional pre-assessment questionnaires (appendix G) and receive some brief psychoeducation on depression as part of the online intervention. Assistance from e-helpers will be available at every stage.

Following initial assessment, participants will then be invited to complete the first session of the intervention online either immediately or in the coming days. E-helpers will attempt to contact participants weekly throughout the intervention in accordance with participant’s contact preferences and following a strict protocol. The guidance will cover areas such as clarifying any difficult concepts, technical support in using the intervention and encouragement in using the materials. Staff providing guidance will keep notes and receive supervision in order to ensure safety and improve quality of the intervention. Participants will complete approximately one session per week either at the public health facility or on their own device elsewhere.

The post-intervention assessment will be scheduled 10 weeks after the pre-intervention assessment, giving participants 10 weeks to complete the 5 week intervention. Participants will also be asked if they would like to fill in an optional user feedback questionnaire. In case participants do not complete the scheduled post-assessment, a maximum of 3 phone call attempts or messages will be made to remind participants to complete the measures. Telephone calls are routinely used in health facilities for care delivery and to contact clients who do not attend. In case no phone number is provided, users will receive three e-mail reminders to complete the questionnaires.

*Participants who Discontinue the Intervention:*

Participants will receive an automated engaging message (either mobile push message or e-mail, depending on whether they use the app or the browser version) when they are inactive for a pre-defined amount of time (e.g. seven days). Three days later they will receive another automated motivating message. If they do not respond, three attempts will be made to establish a personal contact through their preferred method of communication (e-mail, chat, or phone call). Participants who indicate that they wish to discontinue the intervention will be invited to provide their helper with a reason for discontinuation, but will be reminded that this is not required. The purpose of asking these questions is to maximize retention and to learn about barriers for participation.

If participants express that they discontinued the intervention due to heightened distress, appropriate alternative referrals will be discussed with the participant and initiated when necessary. Participants will be asked if they would be willing to be contacted about participation after the intervention for the *Phase 2* Process Evaluation, where we would be asking them for their feedback and experiences of the intervention. As before, it will be made clear to participants that they are free to decline.

*Assessment of Outcomes:*

The measures to be administered are listed in Table 1, details are given of the measures below, and full measures are included in Appendix G. Where local language versions are not available, measure translation will occur alongside intervention translation (van Ommeren at al 1999).

**Table 1: Measures to be used in the e-mental health intervention pilot**

|  | ***Self-Screening*** | ***Pre-treatment measures*** | ***During-treatment measures*** | ***Post-treatment measures*** |
| --- | --- | --- | --- | --- |
| 1. Symptoms of depression | PHQ-9 | PHQ-8 | PHQ-8 | PHQ-8 (primary outcome measure) |
| 1. Functioning | NA | WHODAS 2.0 | NA | WHODAS 2.0 |
| 1. Subjective well-being | NA | WHO-5 | NA | WHO-5 |
| 1. Symptoms of anxiety | NA | GAD7 | NA | GAD7 |
| 1. Self-defined psychosocial goals | NA | PSYCHLOPS | NA | PSYCHLOPS |
| 1. User satisfaction questionnaire | NA | NA | NA | CSQ-8 |

1. PHQ

The PHQ-9 is a well-known 9-item instrument measuring presence and severity of depression (Kroenke et al 2001). PHQ-8 is the same instrument but without the final question on suicidal thoughts. Major depression is diagnosed if 5 or more of the 9 depressive symptom criteria have been present at least “more than half the days” in the past 2 weeks, and 1 of the symptoms is depressed mood or anhedonia. Other depression is diagnosed if 2, 3, or 4 depressive symptoms have been present at least “more than half the days” in the past 2 weeks, and 1 of the symptoms is depressed mood or anhedonia.

As a severity measure, the PHQ-9 score may range from 0 to 27, since each of the 9 items can be scored from 0 (not at all) to 3 (nearly every day). The PHQ-9 has been validated in the Lebanese population with a cut off score of 10 or above indicating probable depression (Sawaya et al 2016). PHQ-8 is the primary outcome measure in this study. The PHQ-8 was chosen because asking about suicide each week could be unduly intrusive given that in normal clinical care, this is not a question that one asks every week. It should be noted that participants at high risk of suicide would have been excluded at the start of the intervention. The research team felt that on balance, it was preferable to monitor changes in depression symptoms using the PHQ-8 (which does not have the suicide item), where any substantial change or increase would result in a e-helper discussing the change with the participant.

1. WHODAS 2.0: socio-demographic information and disability

Data on socio-demographic information (sex, age, education, marital status and work status) will be collected through questions A1-A5 of the 12 item self-report version of the WHO Disability Assessment Schedule 2.0 (WHODAS, WHO 2010), which will be administered first. The WHODAS is a generic assessment instrument assessing health and disability. It is used across all diseases, including mental, neurological and substance use disorders and in many global regions. It is simple to administer, applicable across cultures and can be used in all adult populations. WHODAS covers six domains (cognition, mobility, self-care, getting along, life activities, participation). It assesses difficulties people have across these domains during the last 30 days. Difficulties are scored as none, mild, moderate, severe, or extreme.

1. WHO-5

The WHO-5 Wellbeing Index is a 5-item questionnaire measuring current psychological wellbeing and quality of life, rather than psychopathology (Bech et al 2003). Scores range from 0-25. The scale has demonstrated sensitivity to change in wellbeing and is available in multiple languages.

1. GAD7

The GAD7 is a seven item self-report questionnaire for generalised anxiety disorder (Spitzer et al 2006) widely used in primary and specialist care as an indicator of anxiety symptoms. It consists of likert scale questions including items on nervousness, anxiety, restlessness and fear. It is being included in this study as a means to investigate whether the intervention, which includes a stress reduction exercise and cognitive coping strategies, may reduce comorbid symptoms of anxiety. The GAD7 has been validated in the Lebanese population with an optimum cut off of 10 (Sawaya et al 2016).

1. PSYCHLOPS

The Psychological Outcome Profiles instrument (PSYCHLOPS-Robinson et al 2004) consists of four questions. It contains three domains: problems (2 questions), function (1 question) and wellbeing (1 question). Participants are asked to give free text responses to the problem and function domains. Responses are scored on an ordinal six-point scale producing a maximum score of 20 (5 points per question). The pre- and post-therapy versions of PSYCHLOPS consist of the same four questions but the post-therapy version adds an overall evaluation question (determining self-rated outcome ranging from “much better” to “much worse”). PSYCHLOPS has been validated in primary care populations across several countries (Czachowski et al 2001; Héðinsson et al 2012) . It is currently used in WHO studies in Pakistan, Kenya and Uganda.

1. CLIENT SATISFACTION QUESTIONNAIRE

The Client Satisfaction questionnaire (Larsen et al 1979) is an easily scored and administered eight item measure that is designed to measure client satisfaction with mental health services. It includes an additional free response field as well as the eight likert-scale –type questions. This will be offered to participants as an optional questionnaire after they have completed the post-assessment questionnaires.

*Supervision:*

Weekly supervision will be provided to e-helpers by an appropriately qualified, clinical supervisor from MoPH with a good understanding of the e-mental health project to ensure fidelity of guidance provided, and to support helpers. Supervision will involve discussion of difficulties encountered in supporting the users of the intervention, as well as self-care for e-helpers. The local study coordinator will also have regular contact with the e-helpers.

**Risk mitigation phase 1**

Since the e-mental health intervention is non-pharmacological and there is a broad evidence base for its safe use (Ebert et al 2016), it is unlikely that adverse effects due to the intervention will occur in the exploratory pilot study. The weekly telephone or messaging support will be given by trained study staff (e-helpers) each week. The participants will receive questions approximately once a week about their mood when they start each new session (PHQ-8), the scores of which will be accessible to e-helpers in order for depressive symptoms to be discussed with participants and monitored. We plan to closely monitor the safety of the intervention in this uncontrolled pilot, prior to testing the intervention in an RCT in a later phase.

**Study Phase 2: Process evaluation of administering the e-mental health intervention in Lebanon**

**Purpose**

To gain in-depth information on participant satisfaction and experiences of using the intervention, as well as information on its acceptability and feasibility. This information will guide research design prior to conducting a larger RCT and potential wider implementation of the intervention.

**Overview**

This phase consists of key informant interviews with various categories of people: (a) intervention users; (b) the e-helpers who guided the users; (c) other clinical staff and decision makers. Intervention users will be matched to the interviewer by sex. Key informant interviews will last up to one hour and will follow a semi-structured interview guide (see Appendix H for draft, to be finalized following earlier phases of the intervention, given our iterative process of adaptation and qualitative enquiry). If more time is needed because the respondent has more to say on the subject, subsequent interviews will be agreed with respondents. Details of the interviews are given below.

Informed consent will be obtained prior to interviews (see appendix I). Interviews will be conducted no longer than 6 weeks after the completion of final outcome assessments.

All key informant data will be translated, and analysed following thematic analysis. Findings from this phase of the study will be used to further refine intervention delivery to the local context where required, and to inform the future proposed RCTs of intervention effectiveness.

**Process evaluation:**

*Intervention users*

The sample will consist of approximately 2-4 participants who completed the intervention and approximately 2-4 participants who commenced the intervention but dropped out. These drop-out participants are defined as having completed fewer than 4 online sessions (80%) of intervention following initial consent to participate in the study. They will be clearly informed that participation in subsequent phases of the study is entirely voluntary. For all participants in this qualitative evaluation, we will select based on established methods for qualitative research, such as purposive sampling around certain demographic or participation characteristics to ensure maximum representativeness (e.g. by age/ sex /ethnicity, by those who actively participated in in the intervention versus did not, etc.).

*Process evaluation: E-helpers*

The sample will consist of approximately 2-4 of the e-helpers who guided users through the intervention during the pilot testing phase.

*Process evaluation : Clinical staff and decision makers*

The sample will consist of other cadres of clinical staff/decision makers, such as clinic managers, specialists, NGO managers or district level health systems managers. It is expected they will be recruited through MoPH and other MHPSS organisations.

*Other process measures:*

Through the earlier phases, quantitative data will also be collected with numbers of participants who consent to take part in the intervention, commence the intervention, complete outcome assessments and number of participants who drop-out (along with reasons where given). Additional web analytics (e.g. time spent on web pages, time spent completing sessions) will also be collected.

**Additional Adaptations:**

The research team will review further information gathered from the pilot study and make any necessary adaptations and improvements to the intervention thought to be required. They will record changes made to the interventions in an adaptation monitoring form as a record. The materials will then be prepared for use in subsequent phases of this study (pilot and definitive RCTs and process evaluations) which will be submitted as amendments.

**Risk mitigation phase 2**

Since the e-mental health intervention is non-pharmacological and there is a broad evidence base for its safe use, it is unlikely that adverse effects due to discussing the intervention will occur in during *phase 2*. Nonetheless we plan to closely monitor the safety of the intervention throughout this feasibility pilot, prior to testing the intervention in an RCT in a later phase.

**Study Phase 3: Feasibility RCT to test the procedures of the definitive RCT**

**Purpose**

*Study Phase 3* is a feasibility RCT that precedes the definitive RCT *(Study Phase 5)*, and is intended to test the feasibility and acceptability of the study procedures in the setting in which *Study Phase 5* will be conducted.

**Overview**

*Study Phase 3* will test the relevant processes for a definitive RCT and will be informed by the results of the non-controlled trial and its process evaluation (which is currently still being conducted). This phase will provide further insights on participant recruitment, the screening and randomization procedure, retention, intervention acceptability, training and supervision of staff, protocol adherence, and clinical trial monitoring. The methods applied in this phase are described below. Methods in *Study Phase 5* will be exactly the same; the only difference between the two studies is the sample size. Sample size for the definitive RCT is explained under *Study Phase 5.*

**Participants and procedure**

The study will be carried out in Lebanon under the lead of the MoPH National Mental Health Programme. Participants will include a) Syrian displaced people (n = 30) and b) other groups living in Lebanon (n = 30), and will be recruited through various means including adverts in PHC facilities, community centres, universities and similar, as well as online, through social media and adverts in other forms of media.

At this stage, it will be possible for people interested in participating in the study to access Step-by-Step in two different ways: a) downloading the app to an Android or iOS device, or b) accessing the web version of the application. In *Study Phase 1*, key persons in 22 PHC facilities (e.g. receptionists) were trained in informing attendees about the study and inviting them for participation. Flyers and posters were placed in these facilities, and an introductory movie about Step-by-Step was displayed. However, only a small number of participants were recruited through PHC centres. The majority of participants were recruited via social media. Moreover, the tablets placed in four PHC facilities were not used. NGOs working in the mental health sector, most of them being part of the Mental Health and Psychosocial Support task force, were able to recruit participants through their communication channels, and some of them expressed that their attendees would be interested in using tablets in their facilities. Process evaluation data from *Study Phase 2* will further contribute to fine-tuning the recruitment pathways and the delivery model in the feasibility and definitive RCT.

As in *Study Phase 1,* e-helpers will be trained to provide – mainly through telephone and WhatsApp – standby support, e.g. answering questions on the study and assisting participants with registration, online consent and completion of online questionnaires. The e-helper training will ensure that ethical conduct and study procedures are maintained. E-helpers will also be trained in psychological first aid to respond to acute distress if this should arise during assessments or key informant interviews. The e-helpers will be supervised by the local research coordinator and the clinical supervisor.

Eligible participants are adults (18 years or older) who score 10 or above on the PHQ-9 and above 16 on the WHODAS 2.0. Minors (under the age of 18) and people who have plans to end their life will be excluded from the study (if they answer 2 or higher on the ninth PHQ-9 item and answer “yes” on the additional screening question, see SAFETY CONSIDERATIONS AND FOLLOW UP).

People interested in the study will be provided with all necessary information online. The consent form, informing participants about the study, their participation being voluntary, the two arms, random allocation etc. can be found in Appendix J. After giving consent, participants will be asked to create an account, will first complete the screening questionnaires and (if screened positive) complete the additional baseline questionnaires. Screening questionnaires are used as baseline assessments of depressive symptoms and functional disability. Upon completion of the baseline assessment they will be randomized to either the intervention or ECAU, using a 1:1 allocation ratio.

If the individual does not score over the cut off criteria on the PHQ-9 and WHODAS, information will appear stating that the intervention may not be a good fit and presenting alternative options for receiving general health care and psychosocial support. If the participant confirms plans to end his or her life, an on-screen message will appear explaining that they may need additional mental health support. A national suicide hotline (phone number) was installed by a local NGO in Lebanon which provides 24-hours support and referral to specialised care. People who respond “yes” to the screening question (see SAFETY CONSIDERATIONS AND FOLLOW UP) will be informed about this 24-hours service, along with a list of mhGAP trained PHC facilities, and will be encouraged to seek help. They will not be able to participate in the research.

One-stage stratification will be conducted according to nationality (50% Syrian displaced people and 50% other people residing in Lebanon). As in the pilot trial, people will be asked about their nationality at registration. If they indicated that they are Syrians, they will be asked about how long they have been residing in Lebanon. The study team does not plan to verify the information (e.g. by UNHCR providing information on refugee status), since this would complicate the recruitment process and possibly create suspicion among Syrian displaced people. By installing a procedure for verifying refugee status (or age), we would miss the very important advantage of online and mobile interventions to ensure participants’ privacy. However, we will mention in the limitations section of the paper to be published that all socio-demographic information (as well as all other information) was self-reported and not verified by the study team.

Based on preliminary data from *Study Phase 1*, we anticipate a 50% attrition rate. Since the feasibility RCT will mirror the definitive RCT procedures as closely as possible, stratification will already be done in this study phase. Based on this calculation, 30 Syrians (15 per arm) and 30 Lebanese (15 per arm) will be recruited for the feasibility RCT. Since this is a small feasibility RCT (to inform a definitive RCT) that does not aim to detect statistically significant differences in effectiveness, no power calculations have been carried out.

If randomised into the intervention arm, participants will have access to Step-by-Step until the end of the study. Participants randomised in the control arm will receive basic psychoeducation. Moreover, they will receive a list of selected mhGAP trained PHC facilities with the strong recommendation to seek help in one of these centres (see description of control condition below). The procedures during and after the study (i.e. scheduling sessions, providing guidance) and procedures for the post-intervention assessment will be the same as in *Study Phase 1c),* see page 15*.* Three months after the post-assessment, participants will be asked to complete the follow-up assessment. Participants will be remunerated (approx. $20) upon completion of the follow-up assessment. Different logistical alternatives for this remuneration will be tested in this feasibility trial, e.g. sending phone credit or handing out vouchers for local supermarkets (e.g. through PHC centres).

**Outcomes and measures**

Please note, the following changes to the outcomes and measures are being made for the Phase 5 definitive RCT, they are reported here for ease of reading due to the structure of the protocol.

With Step-by-Step, we aim to detect an effect size of Cohen’s *d* = 0.5 for each of the two primary outcome measures of depressive symptoms (PHQ-9) and functioning (WHODAS 2.0) at post-treatment. Effect size calculation is described on page 26.

The measures used in this study are outlined below, and provided in Appendix K They slightly differ from the measures used in the non-controlled pilot trial: A PTSD Checklist (PCL-6; Lang and Stein 2005) will be added because more than half of the participants will be Syrian displaced people with history of war, displacement and other stressors (Alpak et al 2015). Participants will not be asked about traumatic events, because answering these questions in an app without professional support might be disturbing for them. Additionally, in order to examine cost-effectiveness of Step-by-Step, cost data from an adapted version of the Service Receipt Inventory (SRI; Chisholm, Knapp et al 2000) will be administered (see below). And finally, the PHQ-4 will be used instead of the PHQ-8 in intermediate assessments. The PHQ-4 will no longer be presented when opening a new session, but on a weekly basis regardless of the progress in sessions.

The below matrix gives an overview of the measures in terms of when they will be assessed and what concepts they assess.

**Table 2: Measures to be used in feasibility and the definitive RCT**

|  | ***Self-Screening*** | ***Pre-treatment*** | ***During-treatment*** | ***Post-treatment*** | ***Follow-up*** |
| --- | --- | --- | --- | --- | --- |
| 1. Symptoms of depression | PHQ-9 | NA | PHQ-4 | PHQ-9 (primary outcome measure) | PHQ-9 |
| 1. Functioning | WHODAS 2.0 | NA | NA | WHODAS 2.0  (primary outcome measure) | WHODAS 2.0 |
| 1. Subjective well-being | NA | WHO-5 | NA | WHO-5 | WHO-5 |
| 1. Symptoms of anxiety | NA | GAD7 | NA | GAD7 | GAD7 |
| 1. PCL-5 (eight-items version) | NA | PCL-5 | NA | PCL-5 | PCL-5 |
| 1. Self-defined psychosocial problems | NA | PSYCHLOPS | NA | PSYCHLOPS | PSYCHLOPS |
| 1. User satisfaction questionnaire | NA | NA | NA | CSQ-3 | NA |
| 1. SRI | NA | SRI | NA | SRI | SRI |

1. PHQ-9: Symptoms of depression

Described under *Study Phase 1, part c*, *Assessment of Outcomes*. For the intermediate (i.e., weekly) assessments, the four-items version will be used for reasons of simplicity and engagement and to reduce burden on user. The four-items version has shown good psychometric properties (Kroenke et al., 2009) and the Arabic version was validated among Syrian displaced people in Germany (Kliem et al., 2016).

1. WHODAS 2.0: Functioning

Described under *Study Phase 1, part c*, *Assessment of Outcomes*.

1. WHO-5: Well-being

Described under *Study Phase 1, part c*, *Assessment of Outcomes*.

1. GAD-7: Anxiety

Described under *Study Phase 1, part c*, *Assessment of Outcomes*.

1. PCL-6: PTSD symptoms

PTSD symptoms during the past week will be measured using the abbreviated eight-item version of the PTSD Checklist for DSM-5 (PCL-5; Prince et al., 2016). Items are rated on a five-point scale from 1 to 5 and add up to a total severity score of 30. The previous version of the PCL (PCL-6; Lang and Stein 2005) that was based on the diagnostic criteria of DSM-IV, has shown good psychometric properties and has been tested in diverse cultural settings, including Lebanon (Fares et al 2017).

1. PSYCHLOPS: Self-defined problem(s)

Described under *Study Phase 1c)*, *Assessment of Outcomes*.

1. CSQ-3: User satisfaction

Described under *Study Phase 1c)*, *Assessment of Outcomes*. For reasons of parsimony, we will use the three items version, which uses the most salient items for the measurement of satisfaction with services.

1. SRI: Service utilization

The SRI was developed for the collection of data on service utilization and related characteristics of people with mental disorders, as the basis for calculating the costs of care for mental health cost-effectiveness research (Chisholm, Knapp, et al., 2000). The SRI is currently being adapted for online use and for use in middle-income countries. We will use this adapted version. The SRI will be pilot tested before the feasibility RCT to make sure it is acceptable and feasible.^[[2]](#footnote-3)^

**Interventions**

*Step-by-Step*

The intervention is described on page 10. For the non-controlled pilot study, only an online version (website) was developed. For the feasibility and the definitive RCTs, an iOS/Android app is currently being developed, including some additional features such as notifications or on-board audio recordings. These features will be developed and administered in line with data security regulations (see below). The main advantage of an app is the fact that it can be downloaded once and then used offline, i.e. without Internet connectivity, making participation in the research more equitable for those who cannot afford much mobile data. In addition, an online version (website) will be available, as well, and participants can choose the method of access which best fits their situation.

The delivery model described on page 11 will be fine-tuned depending on the results of the process evaluation in *Study Phase 2*. Preliminary results show that participants used different channels of communication with e-helpers (i.e. telephone, WhatsApp, on-site messaging and e-mail), and some participants did not wish any guidance at all. Therefore, all channels for providing guidance will most likely be maintained (for more information, see Study Phase 5). Furthermore, results showed that the tablets provided to PHC facilities were not used by participants. However, some NGOs working in the mental health sector (and being part of the study, as they collaborate with MoPH and PHC centres) expressed their interest in providing tablets to their attendees for use of the intervention. Therefore, tablets will be made available to facilities by MoPH upon request.

*Enhanced care as usual*

As in previous RCTs on WHO psychological interventions (e.g. Bryant et al 2017, Rahman et al 2016), ECAU will consist of basic psychoeducation and referral to evidence-based care. Since 2015, the MoPH in Lebanon has trained staff in more than 60 PHC centres nationwide on the assessment and management of people with mental health conditions following WHO mhGAP guidelines. Of these trained centres, 42 have received a refresher training and supervision.

If randomised into the control condition, users will first receive basic psychoeducation on depression. The text was taken from the first session of the intervention to make sure the information is identical and be provided via the mobile App / website. Thereafter, users will receive a list indicating selected mhGAP trained PHC facilities where they will receive evidence-based care.

For the cost-effectiveness analysis, service use will be measured with the SRI (see above). These data will be used to estimate how much treatment participants in the control group actually received.

**E-helpers and protocol adherence**

E-helpers are men and women who have a background in psychology, social care or a similar training and will work under the regular supervision of trained clinical psychologists. The recruitment process is done as follows: Job vacancies are posted on the web through universities’ career centers and the MHPSS task force. Applications received are screened and graded by two different persons based on a set of criteria that assesses the relevance of the educational background to health, the relevance of 1 or 2 years of experience in providing psycho social support, maturity level, communication skills, active listening, interest in supporting others, computer skills, language (Arabic and English), and time flexibility. Shortlisted candidates are invited to an interview with the local project coordinator and the operations manager of the National Mental Health Program. Final selection is made after interviewing all eligible candidates.

Prior to the feasibility RCT, they will receive 1-2 weeks of training (to be informed by *Study Phase 1, which is still ongoing*). E-helpers will need to pass a competency test after the training. Only those who pass will be involved in the feasibility (and the definitive) RCT. Those who do not pass will receive additional training and have the chance to repeat the test. During the trial, fidelity checks are conducted to ensure adherence to the guidance protocol. This will be done by the trial monitor – using a treatment fidelity checklist – listening in on a predefined percentage (e.g. 10%) of the guidance calls made during the trial.

**Project monitoring**

As RCTs are logistically complex, a trial monitor will check on a weekly basis that all micro-aspects of this RCT are properly done, in accordance with e- and m-interventions. There will be a weekly, minuted conference call with the local project leadership (local PI, research coordinator, local project manager) and team members based abroad.

**Study Phase 4: Process evaluation of the feasibility RCT**

**Purpose**

To gain in-depth information on participant satisfaction and experiences with using Step-by-Step, and information about the feasibility of the planned RCT procedures, including the recruitment and informed consent procedure, as well as the control condition. This information will mainly inform the definitive RCT.

**Overview**

The feasibility, difficulties and successes in carrying out research and intervention activities will be explored through process monitoring, as well as semi-structured interviews with intervention participants (of both intervention arms), drop-outs, e-helpers, and other key informants.

*Step 1: Process monitoring*

Process monitoring includes review of e-helper records of their 15-min contact with clients and supervision records. Furthermore, data that are automatically collected (e.g. web analytics, number of participants who enrol in intervention, number of dropouts, preferred way of contact, where they heard about the intervention, etc.) will be analysed.

*Step 2: Semi-structured interviews*

Burden of completing the assessments and Step-by-Step on the time and effort of participants, satisfaction with Step-by-Step and ECAU, and barriers and facilitators to adherence will be explored through semi-structured interviews with a sample of 5 participants (including participants that have dropped out). Additionally, interviews will be conducted with e-helpers, clinical supervisors and other key stakeholders to learn about their perspectives. A draft semi-structured interview guide has been developed (see Appendix L) with key questions that are identified for exploration, with additional prompt questions to fully explore each question in depth.

Interviews will follow the same process as outlined for *Study Phase 2*, namely: informed consent will be obtained using the same informed consent procedure as in *Study Phase 2* from all participants immediately prior to interviews (see Appendix I). *Study Phase 2* showed that some users preferred being interviewed via phone because they wished to remain anonymous. Therefore, this option will be given to participants in *Study Phase 4*, as well. No identifying information will be collected during the interview, with all data anonymised. Interviews will be conducted no longer than 4 weeks after the conduct of final outcome assessments, and are expected to last no longer than one hour.

Findings from this phase of the study will be used to further refine recruitment strategies and intervention delivery to the local context where required, as well as to inform the definitive (fully powered) RCT as described in *Study Phase 5*.

**Study Phase 5: Definitive RCT to evaluate effectiveness and cost-effectiveness of Step-by-Step in urban and peri-urban Lebanon**

**Purpose**

To test the effectiveness and cost-effectiveness of Step-by-Step in a fully powered, RCT.

**Overview**

*Study Phase 5* will be carried out in Lebanon under the lead of the MoPH National Mental Health Programme. The methods will mostly be the same as described in *Study Phase 3.* Slight differences in the procedures, based on the experiences from the feasibility RCT, are described below. A refresher training will be done with e-helpers prior to the definitive RCT.

To facilitate the implementation of the trial, and dissemination of the results once the trial has finished, a steering committee with local stakeholders with relevant expertise will be appointed. This steering committee will meet every two months. They will monitor and review trial progress and feedback any concerns or observations to the local Executive Board. The Executive Board consists of members of the National Mental Health Programme, i.e., the Head (Rabih El Chammay), Operations Manager (Perrine Posbic), Service Development Coordinator (Rasha Abi Hana), the local project coordinator (Jinane Abi Ramia), and the trial coordinator (Eva Heim). The Executive Board members are also part of the steering committee.

**Participants and procedure**

This phase of the study will be conducted by the local research team and will follow the same screening and informed consent procedures, inclusion and exclusion criteria as described for the feasibility RCT in *Study Phase 3*. The following changes will be made to the procedures, in accordance with results of the feasibility RCT. These changes mostly relate to automated and other forms of reminders to reduce drop out between the study phases

- In qualitative interviews for the process evaluation (Study Phase 4), users mentioned that the study information was overly lengthy. To address these findings, we decided to develop an animation which explains the most important points in a short video that will be integrated into the app. This should make it easier for participants to understand the information and provide consent.
- Based on qualitative interviews from the uncontrolled pilot trial (Study Phase 2), in which participants mentioned that the story of the intervention was overly lengthy and led to disengagement, we contracted an author to re-write the story to make it shorter, more engaging an to create a third version for people without children. The latter change was done based on feedback from younger participants who were unmarried and could not relate to the character in the story who was married and had children.
- To enhance engagement and reduce the rate of drop out observed between pre-, post- and follow-up assessments in the definitive trial (particularly in the control group), the following measures will be taken to increase retention:
  - At sign-up, users will be asked to choose at least one method of follow up contact from phone call, e-mail, or SMS for reminders of the assessments.
  - Upon sign-up, research staff will either call or message all users in the control group (depending on their preferred contact option) to thank them for their participation in the study and reminding them of the format of the study (e.g. post- and follow up questionnaires).
  - Users who will access the intervention through the app (i.e., not via web-browser) will be asked if they would like to receive push-notifications on their smartphone. These notifications will cover: a) assessments due (both conditions) b) new sessions available (only intervention condition) c) monthly automated messages to remind users of the upcoming assessments and thank them for their ongoing participation in the study assessments (both conditions). Users will receive an explanation at the beginning of the study about the purpose and reasons for these notifications and can opt out at any time after starting the study.
  - If assessments are due, e-helpers will contact users (both intervention and control participants) via their preferred method of contact (phone, email etc).
- For the remuneration for completing all the questionnaires, users will receive $20 phone credit.
- Users will no longer be asked how long they have been residing in Lebanon. This change was made on basis of feedback from refugee organizations in Lebanon.

**Statistical power and sample size**

The power calculation for the definite RCT was based on current literature. A recent meta-analysis of depression treatments in low- and middle-income countries (Cuijpers et al 2018), including different intervention types, formats (e.g. guided self-help, group therapy), and comparators (e.g. waitlist, treatment as usual) identified 32 RCTs. An effect size of 0.73 (Hedge’s *g*) was found for symptoms of depression. Moreover, a recent meta-analysis of Internet- and mobile-based interventions for the treatment of depression in high-income countries, including 19 RCTs, showed an effect size of 0.90 (Hedge’s *g*) when comparing these treatments with waitlist condition.

Despite these relatively high effect sizes, we opt for a more conservative (but still clinically significant) effect size of Cohen’s *d* = 0.5, because of a number of reasons. Firstly, while the large effect size of 0.90 was found in high income countries, internet interventions have been rarely explored in low and middle income country settings (Arjadi et al 2015), thus effects cannot be estimated based on evidence. Secondly, heterogeneity was very high in Cuijper et al’s (2018) meta-analysis which may be explained by the variation in quality of ‘care as usual’ across the study sites, with better care leading to lower effect sizes. Since Lebanon has heavily invested in up-scaling mental health services and training PHC facilities, a lower effect size might be expected. However, whilst services have been improved, they remain limited across the country and further innovative interventions (e.g. online guided self help) are required.

Assuming a power of 0.90 and an alpha of 0.05, the definitive trial needs to have 85 Syrian displaced people and 85 other people residing in Lebanon per arm. After adjusting for 50% attrition, we first calculated that the definitive trial will need to recruit 340 Syrian displaced people and 340 other people residing in Lebanon. However, based on the feasibility trial, a dropout rate of 70% in both groups is expected, such a high dropout is consistent with e-mental health studies (e.g. Van Ballegooijen et al. 2014, Melville et al. 2010). This means that 568 Syrian displaced people and 568 other people residing in Lebanon will be recruited for the definitive trial. Because the Government of Lebanon requires that services - including research on interventions - with Syrian refugees do not exclude the local population, recruitment into both trials will continue until the sample size of both trials is achieved. Analysis will continue to follow the procedures outlines in the section on *data management and statistical analysis* below.

**Outcomes and measures**

The definitive RCT has a t0 and t1 (8 weeks after t0) and an additional follow-up t2 at 3 months after t1, i.e. 5 months after t0. The same measures will be used as in the feasibility RCT. See matrix under *Study Phase 3* for full details.

**The interventions**

The Step-by-Step intervention is described on page 10 and under *Study Phase 3*. ECAU is described under *Study Phase 3*, as well. Two important changes were done to the intervention:

- - While in the previous version of Step-by-Step, only the story was audio recorded, all components of the intervention (i.e., onboarding, questionnaires, interactive elements) will be accessible as audios, primarily with the aim of facilitating the use for people with low literacy levels.
  - The previous exercise “my strengths” was omitted because the uncontrolled pilot study showed that listing one’s own strengths was difficult for participants. The “my strengths” exercise was replaced with a gratitude list, in which users list small things they are thankful for. Evidence indicates that such an exercise might have positive effects on well-being, but we use it rather to increase engagement at the beginning of the intervention than for therapeutic purposes.

**E-helpers and protocol adherence**

The same procedures to ensure protocol adherence will be used as in *Study Phase 3.* However, the number of the fidelity checks was reduced to 5% for two reasons. First, the main fidelity of the intervention lies within the Step-by-Step intervention itself, as all texts and illustrations are standardised. E-helpers provide minimal guidance, which means that they do not provide the techniques to users themselves. Second, due to the re-calculated power analysis, the number of contacts provided by e-helpers is predicted to increase (approx. 2200 contacts in total) which means that with fidelity checks of 5% of the contacts, 110 fidelity checks will be completed, which for minimal guidance is sufficient. In addition, the clinical supervisor will provide ongoing support and training to e-helpers, to make sure they maintain the quality of their support provided to users.

**Project monitoring**

The same procedures of project monitoring will be used as in *Study Phase 3.*

**Study Phase 6: Process evaluation of the definitive RCT**

**Purpose**

To gain in-depth information on participant satisfaction and experiences of using Step-by-Step, further information on its acceptability and feasibility, and information on how it can be integrated into the mental health system (if proven to be effective). This information will mainly inform the future scale-up of Step-by-Step.

**Overview**

Barriers and facilitators of scaling-up Step-by-Step will be explored through comprehensive process monitoring, as well as semi-structured interviews with intervention participants (of both intervention arms), drop-outs, e-helpers, supervisors and other key informants.

*Step 1: Process monitoring*

The same procedures as in *Study Phase 4* will be used.

*Step 2: Semi-structured interviews*

Satisfaction with Step-by-Step, barriers and facilitators to adherence, and relevant information for scale-up will be explored through semi-structured interviews with a sample of 20 participants (ten per arm, including participants that have dropped out). Additionally, interviews will be conducted with e-helpers, supervisors and other key stakeholders such as clinic managers, mental health specialists, MoPH decision makers etc. (up to ten of each group) to learn about their perspectives. For interviews with participants, a reduced version of the interview guide described in *Study Phase 4* will be used. Interviews will follow the same process as outlined for *Study Phase 4* (e.g. giving the possibility to conduct interviews via phone). Interviews with e-helpers and supervisors will follow the procedures in *Study Phase 4*, as well. For interviews with key stakeholders, a draft interview guide is provided in Appendix L.

**SAFETY CONSIDERATIONS AND FOLLOW UP**

The intervention is based on evidence-based therapeutic techniques that have been found to be safe for use in a range of populations, therefore we believe that it is unlikely that distress will increase as a result of participation in the programme. E-helpers will have access to the weekly depression scores of the participants and can therefore monitor distress levels over the course of the intervention.

*Study Phase 1, 3,* and *5:*

All involved research staff will be trained in communication skills, providing support, responding to distress (psychological first aid) and procedures for adverse events including referral procedures. Any adverse or serious events along with actions taken will be documented. All e-helpers will monitor the participant’s weekly depression level using PHQ-4 results.

If, during the course of self-screening or treatment, an adverse event should occur (e.g. the participant discloses plans to end their life or there is a serious protection concern requiring assistance), participants will be provided with information on what services to access following standardised procedures and encouraged to seek support (see below).
 In Study Phase 1, the user will be presented with a PHQ-9 questionnaire at self-screening. If a user should answer affirmatively to item 9 (“thoughts that you were better off dead or hurting yourself in some way”) they will receive another two questions to ascertain whether they have current plans of suicide (see the suicide assessment in Appendix F).

In *Study Phases 3* and *5,* users will receive one additional question only (“In the past month, have you had serious thoughts or a plan to end your life?”), answered with either “Yes” or “No”. If the user is screened in (i.e., answer “no” on the additional question), but expressed suicidal thoughts in the PHQ-9 question, users will be flagged by the system to make sure e-helpers are aware that they have to pay particular attention to any hint to potential suicidal thoughts.

If the participant answers affirmatively to the suicidality screening question, an on-screen message will appear containing the following self-care tips:

- Talk to a trusted family member, friend, or colleague about how you feel.
- If you think you are in immediate danger of harming yourself contact the emergency services or go there directly.
- Call the Embrace lifeline, a hotline established by the Ministry of Public Health for suicide prevention.
- Talk to a professional, such as a doctor, mental health professional, counsellor or social worker.
- If you practice a religion, talk to someone from your religious community who you trust.
- Remember: If you feel like life is not worth living, reach out for help. You are not alone.

And in addition, the participant will be advised to get help and provided with:

- a list of emergency services and PHCs that have received mhGAP-training

The study funds will contribute to the hospital bill for acute treatment and will cover transport costs to the nearest hospital or health facility. The participant will not be able to enter the study.

If a person should disclose an imminent risk of suicide at any point during the intervention, the participant will be provided with details of services that can help, and will be encouraged to get help. Study funds cover transport to the health facility and contribute to costs for hospitalisation.

Participants who disclose an imminent risk of suicide during the study will still be able to take part in the study and receive weekly support, but their data will not be used in analysis. The e-helper will continue to ask about suicidal ideation during every contact thereafter. Moreover, these participants will be called by the clinical supervisor. This plan for risk management is consistent with routine practice in internet intervention research which often offer a crisis support number and advice to seek help (Renton et al 2014).

*Study Phases 2, 4, and 6:*

We foresee no particular risks for participants in these key informant interviews, though interviewers will be trained in responding to participant distress as a result of interview questions. If any distress should arise, immediate referral to supervisors will be made, who will refer the participant to specialist clinical staff for additional support.

**Adverse reactions:**

Although very unlikely, all adverse reactions and serious adverse events ([S]AEs) reported spontaneously by the participant or identified through study measures at any time will be recorded by the e-helpers and escalated to the research coordinator for immediate action and resolution where required.

Detailed procedures for e-helpers to deal with potential (S)AEs were developed by the local study team. These procedures are in line with the national Standard Operational Procedures for dealing with child protection, sexual and gender-based violence, and imminent risk of death or suicide, developed by the Ministry of Public Health.

All SAEs will be reported to the local ethical review committee (ERC) as soon as possible (normally within 24 hours, on working days), and a summary of AEs will be reported to the local ERC every three months. The local ERC will review any SAEs as soon as possible and the summary of AEs during their regular meetings. They will determine any appropriate action in respect of ongoing study conduct. All SAEs will be reported to the WHO ERC.

All AEs will be followed up by the e-helper and the clinical supervisor on a regular basis (e.g. daily, every 2-3 days). The principal investigator will inform the study participants, the WHO ERC and local ERC if anything occurs, on the basis of which it appears that the disadvantages of the intervention may be significantly greater than was foreseen in the research proposal.

**DATA MANAGEMENT AND STATISTICAL ANALYSIS**

Data will be collected electronically, so it is unlikely that there will be any hardcopies of data. However, should there be any hardcopies of data from the study, they will be safely stored in locked cabinets and all electronic data will be stored on password-protected computers. Participants will manage their own account information, including what personal information they enter. Data collected online will be held securely and separately from study data according to relevant data protection laws (see section on Ensuring Privacy and Confidentiality below).

Because of the nature of this innovative intervention, privacy and security of client data is of paramount importance. Please see page 38 on ensuring privacy and security which covers measures in place to ensure security of data collected online.

The data collected predominantly through computers or devices will be downloaded from the platform and migrated into a data-analytic computer program (e.g., SPSS). Data will only be available to the members of the project group. No attributable data will be used in publications or presentations.

For the non-controlled pilot *(Study Phase 1)*, we plan to run statistical tests to assess pre and post differences in measure scores. Test selection will depend on the distribution of the data, but will likely include effect size calculations and chi squared or t-tests. Additional analyses may be conducted investigating usage variables (e.g. time spent on web pages) in order to better understand how participants used the intervention.

For the feasibility and the definitive RCT *(Study Phases 3* and *5)*, both intention-to-treat analysis (including all randomized participants) and completers’ analyses (per protocol, PP) will be carried out. To measure comparisons at baseline between the two treatment groups, *t*-tests will be conducted for continuous variables and Chi-squared test for categorical ones. We will use multiple imputation to deal with missing data.

First, the mean difference between the two treatment arms at baseline, post-intervention, and (for the definitive RCT only) three-month follow-up is determined. Then, the treatment effect is estimated based on intention-to-treat (ITT), using regression estimation models with the principal predictor being treatment assignment status. Missing outcome observations for participants are imputed using multiple imputation exploiting pre-scores and a set of pre-specified background characteristics (gender, age, education, and severity of symptoms). Given that there are two primary outcomes of interest, we impute using multivariate normal regression using an iterative Markov Chain Monte Carlo (MCMC) method based on initial treatment assignment. The aforementioned pre-specified covariates and baseline measurement of primary endpoint are added to the baseline model for improved precision. Potential bias concerns as a result of non-random missing outcome observations are addressed by estimating Lee bounds. Then, 95% confidence intervals are constructed, both for the regression-generated point estimates and Lee (2009) bounds interval estimates.

These treatment effect analyses are performed for both primary outcome measures PHQ-9 and WHODAS 2.0. Concerns of multiple testing error are addressed by maintaining an experiment-wise type I error of 5%. In order to address potential heterogeneity, treatment effects are estimated for sub-groups (e.g. based on pre-scores). Finally, average treatment effects on the treated (ATT) are estimated treatment and corresponding measures of clinically meaningful change and numbers needed to treat (using the approach in Furukuwa et al., 2011) are explored.

In addition, the same analyses will be carried out for analyzing the following clinical outcomes measured at each assessment time: anxiety (GAD-7), well-being (WHO5), posttraumatic stress reactions (PCL-5, eight-items version) and self-identified symptoms (PSYCHLOPS).

Health economic analysis will be conducted to determine the difference in costs and outcomes in the intervention arm as compared to the treatment-as-usual group. Primary analysis will be the total costs over the treatment period and the three months follow-up. Costs include e.g. costs for hosting and maintaining the intervention, costs for e-helpers, and participants’ service use as assessed with the SRI. Between-group comparison of mean costs will be completed using standard *t*-test with ordinary least squares regression used for adjusted analysis, with the validity of results confirmed using bootstrapping.

For the qualitative research in *Study Phases 2, 4 and 6*, data analysis will follow thematic analyses. Sample sizes for qualitative data have been proposed based on previous experience of the number of participants needed to reach saturation. However, where possible a sample-to-saturation approach will be taken, so key informant interviews in *Study Phases 2, 4,* and *6* will be continued until no new substantive information pertaining to the particular topic is raised in subsequent interviews. Analysis of qualitative data will be conducted on translated data, for feasibility reasons.

*For Study Phases 1 and 3*, the sample size was not determined based on the number needed to detect significant change, but rather it was estimated on the ability of health facilities to recruit people and obtain sufficient information. Given the characteristically high drop-out rates associated with psychological interventions, we must ensure enough participants complete the intervention to inform the feasibility and suitability of our research and intervention methodology, including delivery methods, assessment tools, recruitment procedures, and study management.

For *Study Phase 5*, power calculation was done to determine the adequate sample size to detect statistically and clinically significant change.

**QUALITY ASSURANCE**

Participants in *Study Phases 1, 3, and 5* may directly benefit from their participation in the e-mental health intervention, which is based on behavioural activation techniques that are empirically supported. Their participation will furthermore inform local use of such materials, and thus improve knowledge about delivering mental health interventions in the study area. Since the intervention is non-pharmacological and there is a broad evidence base for its use, it is unlikely that adverse effects due to the intervention will occur in in any of the phases of the study. Therefore, we believe that it is not necessary to install a Data Monitoring Safety Board for this study.

**EXPECTED OUTCOMES OF THE STUDY**

Step-by-Step is a potentially scalable psychosocial intervention that requires minimal specialist input, making it potentially particularly useful and appropriate for use in resource-restricted settings where access to mental health care may be severely limited or where stigma prevents people from using services. Feasibility of delivery, safety, acceptability, effectiveness and cost-effectiveness of such an intervention should be first assessed in a setting where research can safely and efficiently be conducted and monitored.

**Expected outcomes of this study include:**

- We expect that the current study will advance knowledge about the feasibility and acceptability of implementing e-mental health in a middle income country, where there is scarce scientific literature.
- The research will further strengthen capacity of the MoPH Lebanon and other project partners in conducting mixed-methods research.
- The definitive RCT will contribute to determine whether the intervention package reduces symptoms of depression, functional disability, anxiety, symptoms of post-traumatic stress and self-identified psychosocial problems and increases well-being among people with depression in Lebanon.
- Pending results of the feasibility and the definitive RCT, the culturally-adapted version of Step-by-Step for Lebanese residents would be finalised and published by WHO and partners, and made freely available. Because this is designed to be suitable for Palestinian and Syrian populations in Lebanon, it is possible that this could be disseminated in other places too.
- The expected longer-term impact is to provide an evidence-based low-intensity, scalable self-help intervention package that may be culturally adapted and implemented in areas affected by adversity or where mental health care is otherwise limited, thereby making important progress in addressing the pertinent issues of improving access and scalability for interventions in hard-to-reach settings.
- Availability of a first-line low-cost treatment package has the potential to increase the number of individuals reached and reduce the burden on more resource-intensive services where available (c.f. stepped care models). Improving access to effective services has the potential to improve people’s psychological wellbeing and functioning.
- If proven effective, a generic version of the e-mental health intervention (with accompanying training materials) will be available for free and by request from WHO with guidance on adaptation, a detailed software user guide and data protection guide. Dissemination of these materials and implementation guides through WHO reduces the gap between an emerging evidence-base and actual service delivery.

**DISSEMINATION OF RESULTS AND PUBLICATION POLICY**

**Local Community**

Members of the local community will be involved in the process evaluation phase. Further community consultations may be used when identified as necessary by the local research team. These might include meetings of experts and interested professionals or community leaders and community members. Where interest in the intervention is high, community meetings can be arranged where results will be communicated.

**Global mental health community**

We will continue discussions with other agencies and research teams throughout this piloting project for further potential test sites. Regular feedback and liaison will be maintained with the appropriate coordination mechanisms for mental health services in Lebanon. This will increase the likelihood that the programme will be able to be imbedded in existing services, increase uptake and sustainability following the research project.

**Academic and Broader Dissemination**

Academic articles will be drafted and submitted to leading journals with broad dissemination. We plan to publish 1) a concept paper describing the intervention itself (published, Carswell et al., 2018), 2) a paper describing the results of the non-controlled pilot study (submitted), 3) a paper describing the results of the feasibility RCT, 4) a paper on the definitive RCT protocol, 5) a paper describing the effectiveness of Step-by-Step in a leading international peer-reviewed journal, and 6) a paper describing the cost-effectiveness of Step-by-Step in a leading international peer-reviewed journal. Academic conference presentations will be given as well. Broad dissemination of WHO publications and tools tends to be excellent, especially in global mental health. WHO therefore has a responsibility to ensure that it releases only high quality and validated materials suitable for wide uptake. New WHO materials need to be tested to ensure effectiveness prior to dissemination.

**DURATION OF THE PROJECT**

The project timeline was adjusted. Study Phases 1 and 2 confirmed the need for a hybrid app and website platform. Unforeseen difficulties in contracting for and developing a bug-free intervention platform (a hybrid app and website platform) caused a delay of several months. Furthermore, Study Phases 1 and 2 showed need for an additional story in the Step-by-Step intervention targeting unmarried people as well as a rewrite of the story (with newly designed images), which has required time.

| **Study Phase 1: Exploratory (non-controlled) pilot study** | |
| --- | --- |
| Preparatory work including training guide production and training staff | Months 1-2 |
| Self-screening of applicants | Months 3-5 |
| Collection of baseline measures | Months 3-5 |
| Conduct intervention | Months 3-8 |
| Collect post-intervention measures | Months 5-10 |
| Data analysis | Month 11 |
| **Study Phase 2: Process evaluation** | |
| Key informant interviews completed post-intervention | Months 10-11 |
| Data entry and analysis | Month 12 |
| **Study Phase 3: Feasibility RCT** | |
| Programming app, developing and adapting new story lines | Months 13-21 |
| Study preparation including development of Standard Operating Procedures, recruiting and training e-helpers, setting up community recruitment channels | Months 13-21 |
| SRI adaptation for Lebanon and pilot testing | Months 13-21 |
| Recruitment and collection of baseline data | Months 22-24 |
| Collection of post-intervention data | Months 24-26 |
| Collection of follow-up intervention data | Months 25-29 |
| Data entry and analysis | Month 30 |
| Submission of feasibility study results to peer-reviewed journal | Month 31 |
| **Study Phase 4: Process evaluation** | |
| Key informant interviews | Month 25 |
| Data entry and analysis | Month 25 |
| **Study Phase 5: Definitive RCT** | |
| Adaptations to the intervention and procedures based on qualitative data following pilot trial | Month 26 |
| Feasibility RCT review / definitive trial preparation meeting | Month 26 |
| Submission of trial protocol paper for publication | Month 27 |
| Refresher training: research and intervention team | Month 27 |
| Recruitment of participants and collection of baseline data for definitive RCT | Months 28-33 |
| Collection of one-week post-intervention data | Months 30-35 |
| Collection of three-months post-intervention data | Months 31-38 |
| Data entry and analysis | Months 39 |
| Preparation and submission of manuscript on definitive RCT results to peer-reviewed journal | Month 40 |
| **Study Phase 6: Process evaluation** | |
| Key informant interviews | Month 39 |
| Data entry and analysis | Month 40 |

**PROBLEMS ANTICIPATED**

While we understand that challenges exist when doing research in areas affected by adversity and limited resources, the collaborators and their networks are experienced in conducting research across Lebanon. The Lebanon study team are all within driving distance of the research areas. Our main project partners, the MoPH Lebanon, are working with the health facilities that will be involved, and therefore have good access to the clinics. Although theoretically there should be less stigma associated to using an e-mental health intervention, stigma may still lead to some people not wanting to take part in the study. Drop-out is a real problem in psychological therapies research, and particularly in e- and m-mental health, so the evaluation phase will help us to understand if drop-out does occur in this feasibility testing.

We anticipate problems with recruiting a large number of participants, particularly Syrian displaced people. In the uncontrolled pilot, the majority of participants were Lebanese nationals. Displaced people are often confronted with many different kinds of problems, and the prospect of receiving an intervention with 50% chance might keep them from giving their consent in participating in the study. Lessons learnt from *Study Phases 1 and 3* will be used to fine tune the recruitment strategies, and especially to address Syrian displaced people. Strong collaborations exist with different partners in the field in order to achieve the proposed sample size.

**PROJECT MANAGEMENT**

Dr Mark van Ommeren and Dr Rabih El Chammay will co-lead the research team, where Dr van Ommeren as the WHO responsible officer will focus more on overall management and design of the study and Dr El Chammay, as the local principal investigator, will focus on design and implementation nationally. Dr van Ommeren and Dr El Chammay have a history of collaboration over the last 5 years. Each will work with a consultant who will specifically assist with project coordination (one at WHO HQ and one at the MoPH in Lebanon) as well as other technical advisors from their working teams. They will consult each other weekly by phone. A local trial coordinator and a trial manager will be contracted for *Study Phases 3-6.*

**ETHICS**

**Involvement of minors**

No minors will be involved in this study. All participants will be adults older than 18 years.

**Ensuring Informed Consent**

All research participants will be asked for individual informed consent, in all phases of the project. A known challenge in informed consent procedures in resource pressured settings is applicants feeling forced to answer assessment questions because the participant sees the research process as an access route to services and other resources. This problem will be diminished by the fact that we will mainly recruit through social and mass media. All participants will go to the site of their own accord and be provided with detailed information at the commencement of the intervention about what they can expect, and reminded that they are free to withdraw at any time and that non participation will not affect in any way their access to their usual health care.

As described throughout the protocol, e-helpers will receive in-depth training on the informed consent process, and will be working in accordance with this protocol. All data and informed consent will be collected online, with telephone or messaging support from e-helpers if necessary. Full information on the study will be provided in local language as part of the consent form before requesting consent, available both written and as audio file. Respondents who decide to participate will be asked to electronically sign the consent form. Because the intervention contains written content (by its nature), inclusion criteria includes that the participant is able to read online intervention content and consent forms, which have been updated with approximately primary school level literacy in mind and to be more in line with WHO ERC-accepted protocol RPC-756.

**Ensuring Privacy and Confidentiality**

As described throughout, and in informed consent forms, participant confidentiality will be protected at all times. All members of the research team will be required to maintain participant confidentiality, with e-helpers receiving training on this as well as ensuring privacy when providing support during the intervention. Process evaluation interviews will all be conducted over the phone, which ensures maximum confidentiality. Calls will be recorded but recordings will be deleted directly after transcription of the interviews. In the case of a participant requiring specialist mental health care due to imminent risk of suicide or other reasons, e-helpers will be trained in the appropriate steps to take to maximise confidentiality, whilst still protecting participant safety and ensuring that adequate care is provided.

Step-by-Step, as any other e-health intervention, carries risk in terms of data protection. In selecting partner institutions to manage the software engineering component of the intervention, potential partners were specifically judged on their data protection system.

***First online-version of Step-by-Step***

The first online version used in the non-controlled trial was programmed by a team at University of Zurich who has over ten years of experience in providing e-health services and uses servers with the highest possible level of data encryption, storage and protection. The server and network infrastructure is operated in the high security Inter Xion Data Center in Zurich. Servers are located in Zurich and thus under lie Swiss data protection laws. The Inter Xion Data Center is ISO27001 certified and complies with the Swiss Financial Market Supervisory Authority (FINMA) guidelines. Secure Sockets Layer (SSL) Certificates can be installed via server administration tools. Data exchanged between the web browser and the web server will be SSL encrypted. Viruses and Trojans are filtered from email and File Transfer Protocol uploads inside a quarantine which also contributes to data protection. A web application firewall filters URL retrievals containing malicious code, preventing unauthorized intrusion into files.

The common procedure with international e-health research is to comply with the data protection laws of the country or continent that are most stringent. In this case, we are obliged to adhere to Swiss law and we will adhere to the Directive95/46/EC of the EU on the protection of individuals with regard to the processing of personal data and on the free movement of such data (http://eur-lex.europa.eu/legal-content/EN/TXT/HTML/?uri=CELEX:31995L0046&from=DE).

Additionally, the intervention website is being designed in such a way that it is not necessary to enter personally identifying information online. Should a need for collection of these data arise they will be held securely and separately from study data according to the relevant data protection laws.

***Native iOS/Android App version (with parallel website)***

For *Study Phases 3 and 5* (feasibility and definitive RCT), the intervention is currently re-programmed by the Freie Universität Berlin, in the context of the STRENGTHS project (“STRENGTHS: Scaling up psychological interventions with Syrian Refugees”). This project has received funding from the European Union (EU)’s Horizon 2020 Research and Innovation programme Societal Challenges. Accordingly, the intervention software and all procedures involving the software will be developed in compliance with the EU General Data Protection Regulation (GDPR), the German Data Protection Act (BDSG) and the Berlin Data Protection Act (BlnDSG).

**Reason for conducting the intervention in settings affected by adversity**

To know the feasibility of this intervention program in real-world settings, it must be tested in the type of situations for which it is developed, that is locations affected by adversity and where there are limitations to mental health care coverage. These communities can be affected by poverty, loss, trauma, ongoing security issues or hosting displaced persons. Previous exposure to violence and loss, disruption of family functioning, and a breakdown in norms and traditional community protective mechanisms can put people at higher risk of mental health problems. Step-by-Step is based on evidence-based psychological techniques that have already been proven effective in other settings. Our study is necessary to test whether these evidence-based techniques can be packaged for settings affected by ongoing adversity and for high-risk populations.

**Potential for adverse effects**

One potential risk is that during the e-mental health intervention, participants experience increased psychological distress. Interventions that require participants to consider stressors or symptoms may cause initial increase in distress, which in the vast majority of cases produces therapeutic benefit in the longer term. All participants will be followed up weekly with a secure message or telephone call by their assigned trained e-helper. In order to support this process, every week, participants will be asked to complete a short self-screening questionnaire (PHQ-4) to monitor whether a participants’ levels of distress has changed significantly. The e-helper supporting the individual will have access to results from this questionnaire in order to better support the individual. This is explained in the consent form. The process for participants who indicate some suicidal ideation is explained on page 32 of this document.

We intentionally chose Lebanon as our site for this study, as we have implementing partners in the area allowing for careful implementation of the protocol with sufficient monitoring of safety and access to services if needed. As mentioned, we will provide information on the nearest specialist facility or hospital to provide support and links to other existing services for participants who experience (S)AEs or for participants screened out of the intervention due to suicidality. Transport to this facility will be provided using study funds.

**Stigma**

To reduce the likelihood of participants experiencing stigmatization from participating in the pilot study, anonymity of participants will be protected. The intervention seeks to ensure that it does not stigmatize participants in a number of ways.

1. The intervention is delivered either at a general PHC centre (not a specialist facility) through tablets in a private space (also used for other activities) or through the users’ own device at home or wherever they choose to use it. Should users participate through a facility tablet, the tablet will also have other health promotion materials on it and be available for use by other patients of the service when not in use by study participants.

2. Participants will be given the choice with regard to the way of being contacted (i.e. telephone, text message, in-site messaging, or e-mail). Next to the input field, there will be a line saying to consider whether the phone is shared and the implications of another phone user knowing they are using the intervention.

3. The content will look like an illustrated book embedded within a website, so the mental health nature of the intervention will not be immediately obvious.

4. The intervention seeks to be non-pathologising, it does not involve identifying diagnostic categories or use specific mental health terminology.

**Equity and accessibility to a written intervention**

Lebanon has a high literacy rate (UNESCO figures show approx. 90% literacy), and Syria also has high literacy rates (86%), however, no studies have been conducted to assess illiteracy among displaced populations. We expect that a high proportion of individuals, but not all, will have the literacy level required to use the intervention. The intervention and consent information have been written for people with a lower literacy level, and e-helpers will be available to assist participants with difficulties related to understanding both during recruitment and use of the intervention. We also included audios for all components of the intervention (i.e., onboarding, assessments, story, exercises) to make it more inclusive for people with low literacy levels.

Despite this equity limitation (which has been acknowledged in WHO ERC guidelines on e-mental health), e-mental health still holds tremendous potential as a public mental health intervention, particularly when seen within a wider system of available interventions. Whilst the MSD department view e-mental health interventions as an important response to addressing mental health problems, they are only one method currently being developed and tested by the department. Trials of other interventions (e.g. RPC 627, 656, 705, 758, ERC0002797 and 0002817) are also underway and use delivery methods which do not require reading or availability of a smartphone device. This suite of interventions will provide service managers with a range of feasible and equitable evidence based tools to improve mental health, of which e-mental health is one.

**INFORMED CONSENT FORMS**

Informed consent forms for all phases have been prepared and attached (Appendix J).

#### OTHER SUPPORT FOR THE PROJECT

#### None.

#### COLLABORATION WITH OTHER SCIENTISTS OR RESEARCH INSTITUTIONS

#### The scientists collaborating in this project are all listed in the General Information section of this document.

The project is implemented in collaboration between:

- World Health Organization (WHO),
- Lebanese MoPH, National Mental Health Programme. Implementing partner. The mental health team will be responsible for coordinating the research activities, coordinating referrals to additional services where required, and overseeing other clinical aspects of the intervention.
- Saint-Joseph’s University (USJ), Beirut. USJ will provide a local ethical committee coverage and advice and will also administrate coverage of costs should participants need access to emergency care.
- International Medical Corps - Implementing partner. IMC runs a number of PHC centres and is fully on board with providing training for e-helpers and access to patients. Their primary health care centres are compliant with the WHO mental health Gap Action Programme and have referral networks to protect participants should they need more intensive care plans.

**FUNDING**

Project activities are funded by two funding organizations:

1. Fondation d’Harcourt, an independent, non-profit foundation based in Geneva, Switzerland. They promote national and international projects and partnerships in the fields of mental health, psychosocial support and social development. For more information, please visit <http://www.fondationdharcourt.org/the-foundation>.
2. elrha Research for health in humanitarian crises (R2HC). The R2HC programme is the product of a strategic partnership between the UK Government ( Department for International Development DFID) and the Wellcome Trust, with Elrha overseeing the programme’s execution and management. For more information, please visit: http://www.elrha.org/r2hc/about/

#### LINKS TO OTHER PROJECTS

#### This project stands on its own.

#### CURRICULUM VITAE OF INVESTIGATORS

#### These are attached.

#### OTHER RESEARCH ACTIVITIES OF THE INVESTIGATORS

#### Other research activities by the investigators are summarized in the attached CVs.

#### FINANCING AND INSURANCE

Insurance is not deemed necessary as follow-up care is available gratis within the Lebanese mental health system.

**References**

Abi Ramia, J., Harper Shehadeh, M., Kheir, W., Zoghbi, E., Watts, S., Heim, E., & El Chammay, R. (2019). Community cognitive interviewing to inform local adaptations of an e-mental health intervention in Lebanon. Global Mental Health, 5, e39. doi:10.1017/gmh.2018.29

Alpak G, Unal A, Bulbul F, Sagaltici E, Bez Y, Altindag A, Dalkilic A, Salvas HA (2015). Post-traumatic stress disorder among Syrian refugees in Turkey: A cross-sectional study. International Journal of Psychiatry in Clinical Practice, 19(1), 45-50.

Andersson G, Cuijpers P (2009). Internet-Based and Other Computerized Psychological Treatments for Adult Depression: A Meta-Analysis. Cognitive Behaviour Therapy;38(4),196-205.

Andersson G, Cuijpers P, Carlbring P, Riper H, Hedman E (2014). Guided Internet-based vs. face-to-face cognitive behavior therapy for psychiatric and somatic disorders: a systematic review and meta-analysis. World Psychiatry 13, 288–295.

Andersson, G, Titov, N (2014). Advantages and limitations of Internet-based interventions for common mental disorders. World Psychiatry 13, 4–11.

Andrews G, Cuijpers P, Craske MG, McEvoy P, Titov N. Computer therapy for the anxiety and depressive disorders is effective, acceptable and practical health care: a meta-analysis. PLoS One, 13;5(10):e13196. doi: 10.1371/journal.pone.0013196.

Arjadi, R, Nauta, MH, Chowdhary, N, Bockting, CLH (2015). A systematic review of online interventions for mental health in low and middle income countries: a neglected field. Global Mental Health 2 (e12).

Bech P, Olsen LR, Kjoller M, Rasmussen NK. Measuring well‐being rather than the absence of distress symptoms: A comparison of the SF‐36 Mental Health subscale and the WHO‐Five well‐being scale. Int J Method Psych Res. 2003;12(2):85-91.

Benish, S.G., S. Quintana, and B.E. Wampold, Culturally adapted psychotherapy and the legitimacy of myth: a direct-comparison meta-analysis. Journal of Counseling Psychology, 2011,58(3):279-89.

Berger T, Boettcher H, Caspar G. Internet-based guided self-help for several anxiety disorders: a randomized controlled trial comparing a tailored with a standardized disorder-specific approach Psychotherapy. 2014 Jun;51(2).

Bernal, G. and E. Sáez-Santiago, Culturally centered psychosocial interventions. Journal of Community Psychology, 2006;34(2):121-132.

Bryant, R. A., Schafer, A., Dawson, K. S., Anjuri, D., Mulili, C., Ndogoni, L., . . . van Ommeren, M. Effectiveness of a brief behavioural intervention on psychological distress among women with a history of gender-based violence in urban Kenya: A randomised clinical trial. PLoS Med; 2017;14(8):e1002371.

Burchert, S., Alkneme, M. S., Bird, M., Carswell, K., Cuijpers, P., Hansen, P., . . . Knaevelsrud, C. (2019). User-centered app adaptation of a low-intensity e-mental health intervention for Syrian refugees. Frontiers in Psychiatry, 9, 663.

Carlbring, P, Maurin L, Törngren C, Linna E, Eriksson T, Sparthan E,. Andersson G. Individually-tailored, Internet-based treatment for anxiety disorders: a randomized controlled trial Behav. Res. Ther. 2011. 49 (1) 18–24.

Carswell, K., Harper-Shehadeh, M., Watts, S., van’t Hof, E., Abi Ramia, J., Heim, E., . . . van Ommeren, M. (2018). Step-by-Step: a new WHO digital mental health intervention for depression. mHealth, 4(8).

Castro F, Barrera M, Holleran Steiker LK. Issues and challenges in the design of culturally adapted evidence-based interventions. Annu Rev Clin Psychol 2010;6:213-239.

Castro FG, Barrera M Jr, Martinez CR. The cultural adaptation of prevention interventions: resolving tensions between fidelity and fit. Prev Sci 2004;5:41–45.

Chisholm D, Knapp MR, Knudsen HC, Amaddeo F, Gaite L, van Wijngaarden B. Client Socio-Demographic and Service Receipt Inventory--European Version: development of an instrument for international research. EPSILON Study 5. European Psychiatric Services: Inputs Linked to Outcome Domains and Needs. British Journal of Psychiatry Supplement. 2000(39):s28-33.

Cuijpers P, Turner EH, Koole SL, van Dijke A, Smit F. What is the threshold for a clinically relevant effect? The case of major depressive disorders. Depression and Anxiety. 2014;31(5):374-8.

Czachowski S, Seed P, Schofield P, Ashworth M. Measuring psychological change during cognitive behaviour therapy in primary care: a Polish study using 'PSYCHLOPS' (Psychological Outcome Profiles). PloS one. 2011;6(12):e27378.

Craig P, Dieppe P, Macintyre S, Michie S, Nazareth I, Petticrew M. Developing and evaluating complex interventions: the new Medical Research Council guidance. BMJ. 2008;337:a1655.

Cuijpers, P., Karyotaki, E., Reijnders, M., Purgato, M., & Barbui, C. Psychotherapies for depression in low- and middle-income countries: a meta-analysis. World Psychiatry. 2018; 17(1):90-101.

Cuijpers P, van Straten A, Warmerdam L. Behavioral activation treatments of depression: A meta-analysis. Clinical Psychology Review. 2007; vol 27 (3), 318–326.

Ebert, D.D., Donkin, L., Andersson, G., Andrews, G., Berger, T., Carlbring, P., Rozenthal, A., Choi, I., Laferton, J.A.C., Johansson, R., Kleiboer, A., Lange, A., Lehr, D., Reins, J.A., Funk, B., Newby, J., Perini, S., Riper, H., Ruwaard, J., Sheeber, L., Snoek, F.J., Titov, N., Ünlü Ince, B., van Bastelaar, K., Vernmark, K., van Straten, A., Warmerdam, L., Salsman, N. and Cuijpers, P. Does Internet-based guided-self-help for depression cause harm? An individual participant data meta-analysis on deterioration rates and its moderators in randomized controlled trials. Psychological Medicine. 2016. 46(13), pp. 2679–2693.

Ekers D, Webster L, Van Straten A, Cuijpers P, Richards D, Gilbody S (2014). Behavioural activation for depression; an update of meta-analysis of effectiveness and sub group analysis. Plos One, 9(6): e100100.

Fares J, Gebeily S, Saad M, et al. Post-traumatic stress disorder in adult victims of cluster munitions in Lebanon: a 10-year longitudinal study. BMJ Open 2017;7:e017214. doi: 10.1136/bmjopen-2017-017214.

Ferrari et al (2013). Burden of Depressive Disorders by Country, Sex, Age, and Year: Findings from the Global Burden of Disease Study 2010. Plos Medicine: 10,11.

Furukawa, T. A., & Leucht, S. (2011). How to obtain NNT from Cohen's d: comparison of two methods. *PloS one*, *6*(4), e19070.

Héðinsson H, Kristjánsdóttir H, Ólason DÞ, Sigurðsson JF. A Validation and Replication Study of the Patient-Generated Measure PSYCHLOPS on an Icelandic Clinical Population. Eur J Psychol Assess. 2012;29(2):89-95.

International Telecommunications Union (2017). Country profile: Lebanon. URL: http://www.itu.int/net4/itu-d/icteye/CountryProfile.aspx#ArabStates. Access date 25.01.2018.

Karam, Elie G et al. (2006) Prevalence and treatment of mental disorders in Lebanon: a national epidemiological survey. The Lancet , 367, 1000 – 1006.

Kleiboer A , Donker T , Seekles W , van Straten A, Riper H, Cuijpers P. A randomized controlled trial on the role of support in internet-based problem solving therapy for depression and anxiety. Behaviour Research and Therapy. 2015. 72, 63–71.

Kliem, S., Mößle, T., Klatt, T., Fleischer, S., Kudlacek, D., Kröger, C., . . . Wiltink, J. (2016). Psychometrische Prüfung einer Hocharabischen Übersetzung des PHQ-4 anhand einer repräsentativen Befragung syrischer Geflüchteter. [Psychometric Evaluation of an Arabic Version of the PHQ-4 Based on a Representative Survey of Syrian Refugees]. Psychother Psych Med, 66(09/10), 385-392. doi:10.1055/s-0042-114775

Knaevelsrud, C., Brand, J., Lange, A., Ruwaard, J., & Wagner, B. (2015). Web-based psychotherapy for posttraumatic stress disorder in war-traumatized Arab patients: randomized controlled trial. J Med Internet Res, 17(3), e71.

Königbauer, J., Letsch, J., Doebler, P., Ebert, D. D., & Baumeister, H. Internet- and mobile-based depression interventions for people with diagnosed depression: A systematic review and meta-analysis. Journal of Affective Disorders; 2017;223:28-40.

Kroenke K, Spitzer RL, Williams JBW. The PHQ-9: Validity of a brief depression severity measure. J Gen Intern Med. 2001;16(9):606-613.

Kroenke, K., Spitzer, R. L., Williams, J. B., & Lowe, B. (2009). An ultra-brief screening scale for anxiety and depression: the PHQ-4. Psychosomatics, 50(6), 613-621. doi:10.1176/appi.psy.50.6.613

Lang AJ, Stein MB. An abbreviated PTSD checklist for use as a screening instrument in primary care. Behaviour Research and Therapy. 2005;43(5):585-94.

Larsen, D.L., Attkisson, C.C., Hargreaves, W.A., and Nguyen, T.D. (1979). Assessment of client/patient satisfaction: Development of a general scale, Evaluation and Program Planning, 2, 197-207

Ly KH, Trüschel A, Jarl L, Magnusson S, Windahl T, Johansson R, Carlbring P, Andersson G (2014). Behavioural activation versus mindfulness-based guided self-help treatment administered through a smartphone application: a randomised controlled trial. BMJ Open. 4:e003440 doi:10.1136/bmjopen-2013-003440

Melville, K. M., Casey, L. M., & Kavanagh, D. J. (2010). Dropout from Internet-based treatment for psychological disorders. British Journal of Clinical Psychology, 49(Pt 4), 455-471. doi:10.1348/014466509x472138

Moock j. (2014). Support from the Internet for Individuals with Mental Disorders: Advantages and Disadvantages of e-Mental Health Service Delivery. 2: 65.doi: 10.3389/fpubh.2014.00065

Price, M., Szafranski, D. D., van Stolk-Cooke, K., & Gros, D. F. (2016). Investigation of abbreviated 4 and 8 item versions of the PTSD Checklist 5. Psychiatry Research, 239, 124-130. doi:10.1016/j.psychres.2016.03.014

Rahman, A., Hamdani, S., Awan, N., & et al. Effect of a multicomponent behavioral intervention in adults impaired by psychological distress in a conflict-affected area of pakistan: A randomized clinical trial. JAMA; 2016;316(24):2609-2617.

Rai, A., et al., Understanding Determinants of Consumer Mobile Health Usage Intentions, Assimilation, and Channel Preferences*.* Journal of Medical Internet Research, 2013; 15(8); e149.

Renton T, Tang H, Ennis N, Cusimano MD, Bhalerao S, Schweizer TA, Topolovec-Vranic J

Web-Based Intervention Programs for Depression: A Scoping Review and Evaluation

J Med Internet Res 2014;16(9):e209

Republic of Lebanon Ministry of Public Health. 2015. Mental Health and Substance Use- Prevention, Promotion, and Treatment- Situation Analysis and Strategy for Lebanon 2015-2020. Beirut: Lebanon.

Republic of Lebanon Ministry of Public Health. 2015. A report of the assessment of the mental health system in Lebanon using the World Health Organization - Assessment Instrument for Mental Health Systems (WHO-AIMS). URL: <http://www.moph.gov.lb/Publications/Documents/WHO_AIMS_Lebanon2015.pdf> Accessed date 09.05.16

Robinson S, Godfrey E, Ashworth M, et al. (2004) A client-generated psychometric instrument: The development of 'PSYCHLOPS'. Couns Psychother Res. Vol 4(2):27-31.

Ruzek, J.I., et al., Mobile mental health interventions following war and disaster*.* mHealth; 2016; 2(9).

Sawaya H., Atoui M., Hamadeh AZeinoun P and Nahas Z. (2016). Adaptation and initial validation of the Patient Health Questionnaire – 9 (PHQ-9) and the Generalized Anxiety Disorder – 7 Questionnaire (GAD-7) in an Arabic speaking Lebanese psychiatric outpatient sample. Psychiatry Research. Vol 239: 245–252

Smith, T.B., Rodríguez, M.D., Bernal, G (2011). Culture. Journal of Clinical Psychology 67, 166–175.

Spek V, Cuijpers P, Nyklicek I, Riper H, Keyzer J, Pop V (2007). Internet-based cognitive behaviour therapy for symptoms of depression and anxiety: a meta-analysis. Psychological Medicine;37(319),328.

Spitzer RL, Kroenke K, Williams JBW, Lowe B. A brief measure for assessing generalized anxiety disorder. Arch Inern Med. 2006;166:1092-1097.

UNHCR (2014). 2015 UNHCR country operations profile – Lebanon. URL: http://www.unhcr.org/pages/49e486676.html. Access date: 11.03.15.

UNHCR (2016). Connecting refugees – how internet and mobile connectivity can improve refugee well-being and transform humanitarian action. Geneva: UNHCR.

Van Ballegooijen W, Cuijpers P, Van Straten A, Karyotaki E, Andersson G, Smit JH, Riper H (2014). Adherence to internet-based and face-to-face cognitive behavioural therapy for depression: a meta-analysis. Plos One, 9(7): e100674.

Van’t Hof E, Stein DJ, Marks I, Tomlinson M, Cuijpers P. (2011).The effectiveness of problem solving therapy in deprived South African communities: results from a pilot study. BMC Psychiatry. 30;11:156. doi: 10.1186/1471-244X-11-156.

Van de Vijver FJ, Leung K. Methods and data analysis for cross-cultural research. Vol 1: Sage; 1997.

Wang Z, Wang J, Maercker A. Chinese My Trauma Recovery, a Web-based intervention for traumatized persons in two parallel samples: Randomized controlled trial. J Med Internet Res 2013;15(9):e213.

World Health Organisation. Measuring health and disability: Manual for WHO Disability Assessment Schedule (WHODAS 2.0). 2010; Geneva.

World Health Organization. 16 March 2012. Report by the Secretariat for the Sixty-fifth World Health Assembly (document A65/10). Global burden of mental disorders and the need for a comprehensive coordinated response from health and social sectors at the country level.

World Health Organization (2015). Comparative effectiveness of different formats of psychological treatments for depressive disorder. URL: <http://www.who.int/mental_health/mhgap/evidence/depression/q8/en/>. Accessed 11.03.2016

WHO, *Problem Management Plus (PM+): Individual psychological help for adults impaired by distress in communities exposed to adversity. (Generic field-trial version 1.0).* 2016, Geneva: WHO.

1. Appendix A was updated for version 6 of the protocol. [↑](#footnote-ref-2)
2. The current adaptation of the SRI for use in low- and middle-income countries and for online/app use is done within a large EU funded program, the STRENGTHS programme (<http://strengths-project.eu/en/strengths-home/>). Within this program, the Step-by-Step App and website will be tested in three parallel RCTs in Sweden, Germany and Tunisia. Results of the SRI pilot testing in STRENGTHS will be used for adaptation of the SRI in Lebanon. [↑](#footnote-ref-3)
