# Supplementary material for: Effects of a WHO-guided digital health intervention for depression in Syrian refugees in Lebanon: A randomized controlled trial
Source: PLoS Med. 2022 Jun 23;19(6):e1004025. doi: 10.1371/journal.pmed.1004025 (PMC9223343; doi:10.1371/journal.pmed.1004025)
Supplement: S1 Appendix — (DOCX) [file pmed.1004025.s003.docx]

S1 Appendix: Details about the statistical procedures

In order to explore the potential implications of MNAR, we utilize Random Forest Lee Bounds (RFLB) as a sensitivity analysis, as introduced in Cornelisz et al. (2020) [1]. In assuming MNAR, the RFLB approach addresses differential attrition across treatment arms. In addition to the assumption of successful randomization, it only adds a monotonicity assumption for the selection mechanism of missing outcomes. This assumption entails that assignment to treatment can only affect the likelihood of attrition in one direction, such that there is no heterogeneous effect–in terms of sign- of treatment assignment on attrition. It then trims away observations from the treatment arm suffering from the least attrition in two ways to derive worst- and best-case scenarios.

In this study, attrition is higher for the intervention group. The RFLB procedure in this study thus trims the outcome distribution of the control group by removing observations from the lower (upper) end of the distribution such that an upper (lower) bound for the treatment effect estimate is estimated. For example, attrition for PHQ9 at post-measurement is 49.8% for the intervention group, relative to 37.1% for the control group. The trimming proportion is thus roughly 20.3% (i.e., ((0.498-0.371))⁄((1-0.371))=0.203 ) of the observed control observations. This implies that for PHQ9 at post-measurement the RFLB removes the 37 (i.e., 0.203 * 180 = 37) highest (lowest) observations from the total of 183 observations observed in the control group and compares the resulting mean to that of the intervention group as to get the upper (lower) bounds of the treatment effect estimate. Performing this RFLB approach for the point estimate of the effect on PHQ9 at post-measurement (-2.81) yields a lower bound of -4.87 and an upper bound -0.89, as reported in Table 3.

In sum, the MNAR mechanism imposed by RFLB is quite extreme and, given that we do not observe clear signs of selective attrition on observed covariates, also highly unlikely in the context of this study. As such, the most informative estimates of the true treatment effects for this study are the MI estimates assuming MAR.

Reference

1. Cornelisz I, Cuijpers P, Donker T, van Klaveren C. Addressing missing data in randomized clinical trials: A causal inference perspective. PLoS One 2020;15:e0234349. doi: 10.1371/journal.pone.0234349
